# A standard system phantom for magnetic resonance imaging

**DOI:** 10.1002/mrm.28779

**Published:** 2021-04-13

**Authors:** Karl F. Stupic, Maureen Ainslie, Michael A. Boss, Cecil Charles, Andrew M. Dienstfrey, Jeffrey L. Evelhoch, Paul Finn, Zydrunas Gimbutas, Jeffrey L. Gunter, Derek L. G. Hill, Clifford R. Jack, Edward F. Jackson, Todor Karaulanov, Kathryn E. Keenan, Guoying Liu, Michele N. Martin, Pottumarthi V. Prasad, Nikki S. Rentz, Chun Yuan, Stephen E. Russek

**Affiliations:** ^1^ Physical Measurement Laboratory National Institute of Standards and Technology Boulder Colorado USA; ^2^ Department of Radiology Duke University Durham North Carolina USA; ^3^ American College of Radiology Philadelphia Pennsylvania USA; ^4^ Merck Research Laboratories West Point Pennsylvania USA; ^5^ University of California Los Angeles California USA; ^6^ Department of Radiology Mayo Clinic Rochester Minnesota USA; ^7^ Centre for Medical Image Computing University College London London United Kingdom; ^8^ Medical Physics University of Wisconsin Madison Wisconsin USA; ^9^ CaliberMRI, Inc. Boulder Colorado USA; ^10^ National Institute of Biomedical Imaging and Bioengineering, National Institutes of Health Bethesda Maryland USA; ^11^ Radiology/CAI NorthShore University HealthSystem Evanston Illinois USA; ^12^ Radiology University of Washington Seattle Washington USA

**Keywords:** MRI standards, phantom, quality assurance, quantitative MRI

## Abstract

**Purpose:**

A standard MRI system phantom has been designed and fabricated to assess scanner performance, stability, comparability and assess the accuracy of quantitative relaxation time imaging. The phantom is unique in having traceability to the International System of Units, a high level of precision, and monitoring by a national metrology institute. Here, we describe the phantom design, construction, imaging protocols, and measurement of geometric distortion, resolution, slice profile, signal‐to‐noise ratio (SNR), proton‐spin relaxation times, image uniformity and proton density.

**Methods:**

The system phantom, designed by the International Society of Magnetic Resonance in Medicine ad hoc committee on Standards for Quantitative MR, is a 200 mm spherical structure that contains a 57‐element fiducial array; two relaxation time arrays; a proton density/SNR array; resolution and slice‐profile insets. Standard imaging protocols are presented, which provide rapid assessment of geometric distortion, image uniformity, *T*_1_ and *T*_2_ mapping, image resolution, slice profile, and SNR.

**Results:**

Fiducial array analysis gives assessment of intrinsic geometric distortions, which can vary considerably between scanners and correction techniques. This analysis also measures scanner/coil image uniformity, spatial calibration accuracy, and local volume distortion. An advanced resolution analysis gives both scanner and protocol contributions. SNR analysis gives both temporal and spatial contributions.

**Conclusions:**

A standard system phantom is useful for characterization of scanner performance, monitoring a scanner over time, and to compare different scanners. This type of calibration structure is useful for quality assurance, benchmarking quantitative MRI protocols, and to transition MRI from a qualitative imaging technique to a precise metrology with documented accuracy and uncertainty.

## 1 INTRODUCTION

With the rise of quantitative MRI to complement qualitative MRI, it is imperative to assess system stability and understand the comparability of measurements across MRI scanners. One solution is to use a standard imaging phantom, an inanimate object used to characterize or calibrate an imaging system. A standard phantom would be one that is commonly used and accepted by the imaging community; has stable, precisely defined properties to allow monitoring of scanner performance and accuracy of image‐based measurements; is fully documented; has long‐term monitoring and maintenance; and is traceably connected to the International System of Measurements. The need for a standard MRI system phantom was voiced at a National Institute of Standards and Technology (NIST) workshop entitled “Imaging as a Biomarker: Standards for Change Measurements in Therapy,” that was held on September 14‐15, 2006.^1^ The workshop action items included: (1) Design phantoms that may better characterize the time‐related physical performance of imaging systems, and the performance of specific functional and molecular‐based measurements; (2) Define the physical performance of different imaging platforms required to measure change analysis; and (3) Develop and share open source tools to analyze phantom or simulated data.

The Ad Hoc Committee for Standards in Quantitative Magnetic Resonance (SQMR) was formed, under the auspices of the International Society of Magnetic Resonance in Medicine (ISMRM), to address these action items in the areas that pertained to MRI. The committee developed recommendations for a system phantom^2^ that could be used to determine the accuracy, stability, and comparability of MRI scanners. The desired measurements include:

1. Radio frequency (RF) field, *B*_1_, non‐uniformity
2. Static main magnetic field, *B*_0_, non‐uniformity
3. Geometric linearity
4. Gradient amplitude
5. Slice position and profile
6. Image uniformity
7. Resolution (high‐contrast detectability)
8. Signal‐to‐noise ratio (SNR) (low‐contrast detectability)
9. Accuracy and precision of measurement of proton spin relaxation times: *T*_1_ (longitudinal) and *T*_2_ (transverse) and proton density
10. System constancy

Such a system phantom can be used to: (1) track scanner performance at a particular site over time, as well as compare performance with other scanners; (2) determine the accuracy of certain quantitative measurements, such as *T*_1_ mapping, and assist in the development of appropriate imaging protocols to obtain desired accuracy; (3) validate scanner and protocols for participation in clinical trials; and (4) determine the best phantom fabrication techniques for future phantoms, for example, application‐specific phantoms.

Previous successful efforts to create standard phantoms include the American College of Radiology (ACR) MRI phantom^3^ and the Alzheimer’s Disease Neuroimaging Initiative (ADNI) MRI phantom.^4^ The ACR magnetic resonance accreditation phantom is a water‐filled cylindrical phantom with relaxation times and conductivities in the biological range of interest. The phantom contains a resolution inset, slice profile wedges, a grid to determine geometric distortion, and a contrast array. The phantom was primarily designed as a method to assist site accreditation performed by the ACR and as a mechanism to improve quality control by repeated imaging on a weekly basis. The spherical ADNI phantom is used for measurements of SNR, contrast‐to‐noise ratio (CNR), and geometric distortion in scan protocols intended to monitor changes in the brain morphology over time. From 2004 to 2009, the ADNI study acquired a phantom image with each participant study.^4^ The key results from this phantom plus human‐participant study were: (1) the drift in scanner gradient calibration over months to years was less than the magnitude of step‐wise changes in the gradient calibration introduced by field service calibration; (2) using phantom derived linear scaling corrections was not better than affine registration at correcting within‐subject longitudinal‐imaging‐series gradient calibration changes or gradient drift. For ADNI‐2 (2010‐2017), phantom images were acquired for site certification and after system upgrades. These images were used to ensure that off‐line gradient non‐linearity corrections based on scanner model could be performed correctly.^5^ In ADNI‐3 (2018‐present), all scanners in the study have full three‐dimensional (3D) on‐board corrections for gradient non‐linearity, and sites are requested to use ADNI phantoms for certification if possible. The standard system phantom described here builds off the ACR and ADNI phantoms, addressing the need for a more general and precise calibration object that has properties traceable to primary standards connected to the International System of Units (SI).^2^

This paper describes the design, construction, accuracy requirements, and measurement protocols of prototype system phantoms designed by the ISMRM SQMR ad hoc committee. The system phantom is a precise imaging artifact for quantitatively measuring image distortions and validating many types of quantitative mapping. The system phantom has subsequently been commercialized with over 100 units sold.^6^ The commercial system phantom has some minor changes in its design to accommodate more efficient manufacture, reduce cost, and incorporate some improvements that have been requested by customers. These changes are documented in Supporting Information Sec. 9. Here, we note that the spin‐relaxation times and geometric tolerances of the commercial phantoms vary slightly from the prototypes and calibration data for the particular phantom, as determined from the serial number, should be used. The 3D mechanical design files, analysis software, analysis instructions, and imaging data can be found online, as described in the Data Availability Statement.

A single phantom cannot provide all the calibration measurements required for assessing the accuracy of MRI scanners and quantitative imaging protocols. However, this system phantom can address many of the important measurement and quality control issues. A universally used phantom is essential to allow sites to measure system stability and readily compare scanner performance with other scanners. There are several elements of the MRI system phantom described here that make it unique:

1. SI traceable parameters including geometric dimensions (mm), nuclear magnetic resonance (NMR) parameters such as proton spin relaxation times (s), and material compositions that dictate contrast properties.
2. Dimensionally stable construction using a rigid precision‐joined framework and materials that are both low thermal‐expansion and low water‐uptake.
3. All components including design, 3D models, imaging protocols, detailed description of construction materials, and imaging analysis routines are in the public domain.
4. Long‐term maintenance/monitoring of stability and accuracy by a national metrology institute.

## 2 METHODS

This section gives an overview of the design and construction of prototype MRI system phantoms and the process to provide SI‐traceable calibration of phantom properties. Two prototype system phantoms were built for this study. A more comprehensive description of the construction, solutions, and materials can be found in the Supporting Information. The design and construction specifications are meant as a common reference for commercial versions, not as specific requirements. The prototype phantoms are modular so that all components may be disassembled and reassembled in a variety of configurations for testing alternative designs.

### 2.1 System phantom design

**FIGURE 1:**
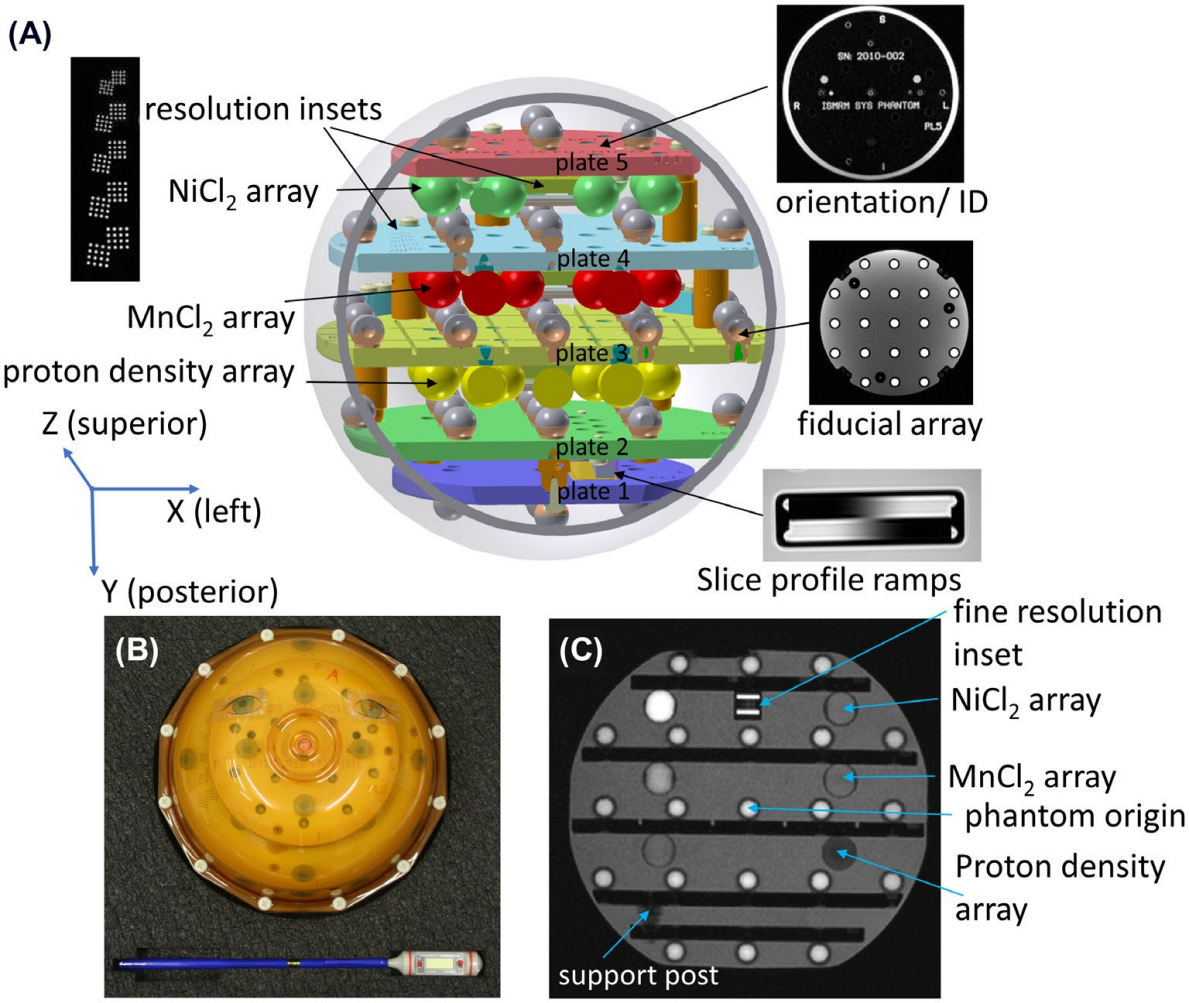
A, Schematic of system phantom showing fiducial array (gray/brown), NiCl_2_ array (green), MnCl_2_ array (red), proton density array (yellow), and resolution and slice profile insets. Plate 5 contains MR visible engravings with the serial number and phantom type. All plates have orientational marks and plate 3 contains a grid for manual estimation of geometric distortion. B, Top view of phantom showing eye decals. C, Sagittal slice in the Y‐Z plane showing the MR parameter arrays, fiducial elements, phantom origin, and the resolution inset.

The prototype MRI system phantom, shown in Figure 1, consists of a water‐filled spherical polycarbonate shell with a 200 mm inner diameter (ID). The spherical diameter was chosen to mimic a human head and was designed to fit in most head coil assemblies. A spherical design also allows for easy rotation of the phantom. Inside the spherical shell is a framework consisting of five 8.0 mm‐thick polyphenylene sulfide (PPS) plates rigidly connected with PPS rods and kinematic mounts. PPS was chosen since it is a high‐performance plastic with low water absorption, low thermal expansion, and good machinability. The PPS plates are annealed for stress relief and then machined to an inspected flatness of less than 0.04 mm. The plates support 57 fiducial spheres, a 14 element NiCl_2_ array, a 14 element MnCl_2_ array, a 14 element proton density array, two resolution insets, and wedges for slice profiles. The plates, as shown in Figure 1, contain the following structures: Plate 1, 5 fiducial spheres on bottom; Plate 2, 13 fiducial spheres on top and slice profile wedges on bottom; Plate 3, 21 fiducial spheres on top, proton density array on bottom; Plate 4, coarse resolution inset in the plate, 13 fiducial spheres on top, MnCl_2_ array on bottom; Plate 5, 5 fiducial spheres on top, serial numbers and phantom ID in the plate, and NiCl_2_ array and fine resolution inset on bottom. The fine resolution inset can also be mounted on plates 3 or 4.

The phantom coordinate system was chosen to match typical scanner/patient coordinates, with +Z along the scanner bore axis (superior), +X toward the left, and +Y down (posterior), when mounting the phantom in the default orientation. A set of eye decals is included on the phantom shell to allow easy recognition and placement of the phantom within the scanner, as seen in Figure 1B. The phantom is placed in the same fashion as a patient laying supine on the imaging bed. The origin of the phantom is defined as the center of the central fiducial sphere as shown in Figure 1C. The phantom is designed so that the plates are in the XZ (coronal) plane with left (L), right (R), superior (S), and inferior (I) marked on each plate, along with the plate identifier. Phantom identification information, along with the serial number, are etched into plate 5, which are easily readable in the prescribed 3D scan (Figure 1A). In the default orientation, the MR parameter arrays (NiCl_2_, MnCl_2_, proton density arrays) are imaged with a coronal scan. Recommended protocols for measuring the arrays and insets on clinical MRI systems, for the main MRI vendors, are given in the Supporting Information, Tables S4 through S10.

The inner frame of the prototype phantoms was designed to be very rigid and allow the fiducial‐sphere centers to be located within a ±0.1 mm accuracy. Details of the plate locating mechanism are shown in Supporting Information Figure S1. A hemispherical machined surface on the post mates with a set of inclined plates to precisely locate the plates and eliminate torsional bending. The precision construction of the prototypes comes at an increased cost due to the need for computer‐controlled machining. It was deemed necessary for the prototypes to have such precision to allow the study of scanner geometric distortion.

### 2.2 Fiducial array

The system phantom contains a 3D fiducial array that can be used to assess geometric distortion, image uniformity and *B*_1_ homogeneity. The fiducial array was modeled on the ADNI phantom,^4^ which has successfully been used to correct geometric distortions and image nonuniformity. The fiducial array spheres all have the same fill solutions with the same *T*_1_ and *T*_2_ properties (*T*_1_ = 407 ms ± 6 ms, *T*_2_ = 347 ms ± 6 ms at 20.0°C, 3.0 T), which means they can be used to assess image homogeneity and calculate *B*_1_ variation, both transmit, $B_{1}^{+}$, and receive, $B_{1}^{‐}$, fields.

**FIGURE 2:**
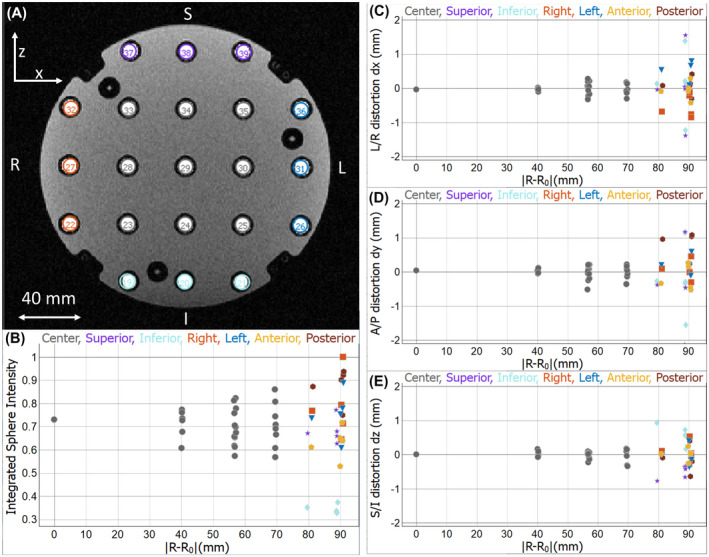
A, Coronal 3D gradient echo image of the fiducial array along with 10.0 mm circular ROIs located on prescribed 40 mm grid, which allows for visual identification of geometric distortion. S, I, L, R refer to superior, inferior, left, right directions, respectively. The scan is at 3T using a body transmit coil and a head receive coil with 0.97 mm isotropic voxels, TR = 6.3 ms, TE = 1.89 ms. The nonuniform gradient corrections are turned off. B, Normalized integrated intensity of all 57 spheres obtained after the sphere location procedure. C,D,E, Geometric distortion along the x, y, z directions, respectively. Geometric distortion, $\delta \vec{R}={\vec{R}}_{a}‐{\vec{R}}_{p}$, is defined as the distance between the apparent position ${\vec{R}}_{a}$ and the prescribed position ${\vec{R}}_{p}$, of the sphere centers after an affine transformation has accounted for rotation, translation, and overall scale correction.

The fiducial array consists of a set of 57 precision‐machined polyvinyl chloride (PVC) 10.0 mm ± 0.1 mm inner‐diameter spheres on a 3D cubic lattice, with a lattice spacing of 40 mm ± 0.1 mm. Figure 2 shows a coronal MRI with a subset of the fiducial spheres along with 10 mm diameter circular regions of interest (ROIs) at their prescribed positions, before automated sphere location. Figure 2 shows standard output from the Python‐based analysis package, after sphere location, plotting image uniformity and geometric distortion along the *x*, *y*, *z* axes, respectively. The fiducial spheres are numbered 1 through 57, starting at plate 1, increasing from right to left, then inferior to superior. For data presentation purposes, the 57 fiducial array elements are divided into seven groups: 27 internal spheres on central 3 × 3 × 3 grid, 5 spheres at each of the six outward faces of the phantom. Each set of fiducials is represented by a different color in the plots in Figure 2.

The spheres are filled with a CuSO_4_ solution made by mixing 0.802 g CuSO_4_* 5H_2_O per liter of deionized water (Supporting Information Sec. 4). The Cu^+2^ ion concentration was measured, using an inductively coupled plasma‐optical emission spectroscopy and a NIST traceable Cu standard (such as NIST standard reference material 3114) to be 0.18901 ± 0.00055 mg/g or 2.969 ± 0.008 mM. A small amount of blue dye (erioglaucine disodium salt, 0.12 µg/ml) was added to assist in the visual recognition of the fiducial elements. The fiducial spheres are machined in two parts from PVC stock, glued together and attached to the plates using an M8‐1.25 threaded stub, as shown in Supporting Information Figure S1C. PVC was chosen because it is inexpensive, can be easily glued, and has a low permeability to water. The spheres are sealed with a tapered polyvinylidene difluoride (PVDF) plug. The fiducial solution was chosen to have short *T*_1_ times to give, for most scans in the protocol, high signal and contrast with respect to the surrounding fill composed of long‐*T*_1_ deionized water. High contrast is beneficial for automated location of the fiducial sphere positions.

### 2.3 MR parameter arrays (NiCl_2_, MnCl_2_, and proton density arrays)

The MR parameter array spheres are 20 mm outer diameter (OD), 15 mm internal diameter (ID) polypropylene spheres that are filled, heat sealed and then, epoxy sealed to an M8‐1.25 threaded mounting stud (Supporting Information Figure S1D), similar to those used for the fiducial spheres. Since the spheres are epoxied onto a hemispherical indent, as opposed to being integrally machined with the threaded stud, there is more positional uncertainty in the MR parameter arrays than in the fiducial array. Polypropylene spheres were chosen since they are inexpensive, commercially available, and have one of the lowest water/air permeation rates among all of the plastics tested. However, the OD, ID, and sphericity of the polypropylene spheres are not well controlled: see Figure S2D inset. The polypropylene spheres are made by thermally welding together two molded hemispheres, which results in a thicker wall in the weld plane.

The *T*_1_ and *T*_2_ values were modified by doping high‐purity water with NiCl_2_ and MnCl_2_ in their respective arrays (Supporting Information Sec. 4). The proton density values were modified by making solutions of 5 % to 100 % high‐purity water with the balance D_2_O. Proton density is defined as the concentration of MR‐visible protons in a sample/tissue, relative to that in the same volume of water at the same temperature.^7^ All solutions use American Chemical Society (ACS) reagent grade,^8^ or higher quality, water. A small quantity of NiCl_2_ (0.26 mg Ni^2+^/ml) was added to the proton density solutions to reduce the relaxation times. Green dye (28 µg/mL erioglaucine disodium salt, 28 µg/mL tartrazine) was added to the NiCl_2_‐doped array solutions, red dye (49.6 µg/mL allure red AC) was added to the MnCl_2_‐doped array solutions, and yellow dye (53 µg/mL tartrazine) was added to the proton density solutions to make the arrays easily identifiable by the user. Each array consists of 14 spheres, 10 equally spaced on a 50 mm radius, and 4 spheres internal to the outside 10 spheres on a 40 mm grid. The sphere positions, NiCl_2_, MnCl_2_, D_2_O concentrations, and measured *T*_1_, *T*_2_ values (at 20°C, 1.5 T and 3.0 T) for the NiCl_2_, MnCl_2_, and proton density arrays are listed in the Supporting Information in Tables S1‐S3, respectively.

**FIGURE 3:**
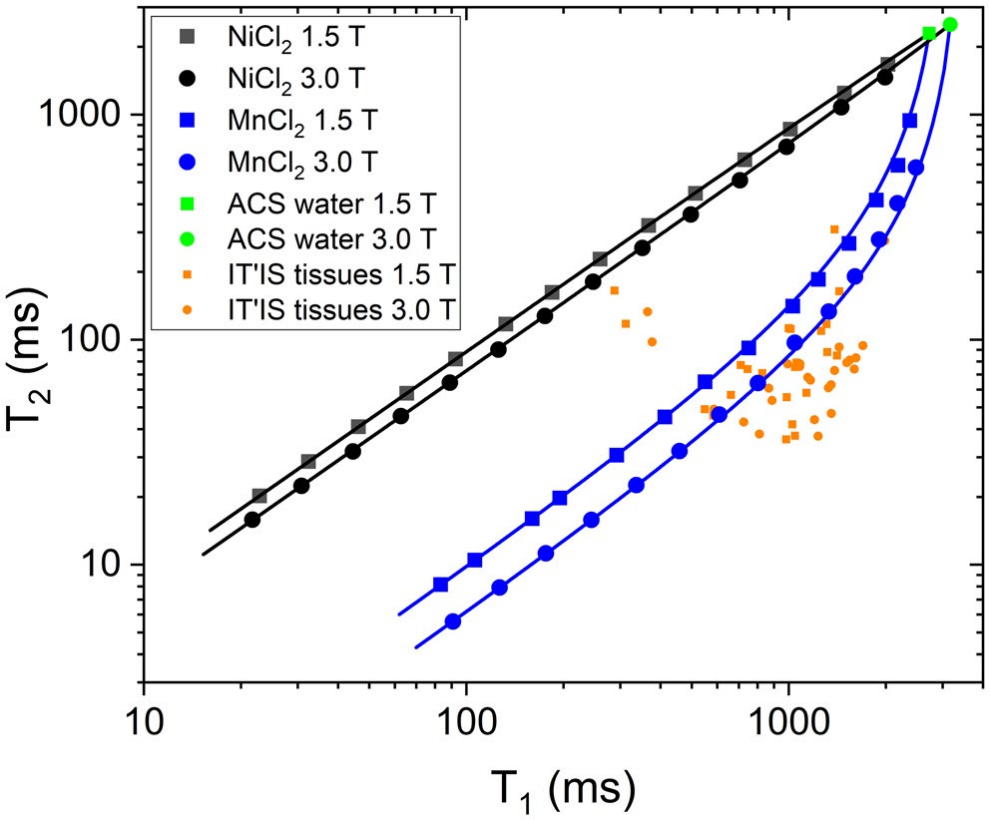
Overview of *T*_1_ and *T*_2_ properties of MR parameter arrays at 20.0°C. 1.5 T data are plotted as circles, 3.0 T data as squares. Also plotted for reference, are values for various human tissues (in orange) at 1.5 T and 3.0 T at 37°C, taken from the online IT’IS data base and values for high purity water at 20°C (in green) The solid lines are model fits assuming that the relaxivities are linearly dependent on the paramagnetic salt concentrations.

The NiCl_2_ and MnCl_2_ arrays were chosen to have a wide range of values to mimic both endogenous tissues and tissue containing standard commercial contrast agents. *T*_1_ and *T*_2_ relaxation times for the arrays, along with typical values for tissue,^9^ are shown in Figure 3. The MnCl_2_‐doped array spans a range of relaxation time values much closer to that of tissue; however, it has considerably more temperature and field dependence than the NiCl_2_‐doped array (Supporting Information Sec. 7). *T*_1_ and *T*_2_ can be measured on both the NiCl_2_‐doped and MnCl_2_‐doped arrays; although historically, the NiCl_2_ array was designed to cover a suitable range of *T*_1_ values, while the MnCl_2_ array was chosen to cover a suitable range of *T*_2_ values. Also shown in Figure 3 are model fits to the data, assuming that the relaxation rates are linearly proportional to the paramagnetic salt concentration. The deviation of the measured data from the model and the relation to the measurement uncertainties are discussed in the Supporting Information Sec 2.

The NiCl_2_ doping concentrations were chosen to give a $\sqrt{2}$ progression of *T*_1_ values from approximately 20 ms to 2000 ms at a *B*_0_ field of 1.5 T. NiCl_2_ was chosen because the relaxivity of paramagnetic spin‐1 Ni^2+^ ions on water proton spins has been extensively studied.^10^, ^11^ *T*_1_ values are relatively insensitive to the magnetic field strength^12^ and to temperature over the range of application for this phantom.^10^ However, the paramagnetic relaxation enhancement due to Ni^2+^ has a complex behavior in the region of application with non‐monatonic variation of the relaxivities with field and temperature.^12^ *T*_1_ and *T*_2_ for the higher concentration NiCl_2_ solutions, Ni‐5 to Ni‐14, have a minimum near 22°C at 3 T (Supporting Information Sec 7), close to typical MRI bore temperatures. The presence of a minimum eliminates the large linear variation of *T*_1_ and *T*_2_ with temperature, giving a maximum variation of 4 % over the range of expected bore temperatures (16°C to 26°C).

The MnCl_2_ concentrations are varied from 0.013 mM to 1.704 mM and, as with the NiCl_2_ array, the concentrations were spaced by factors of $\sqrt{2}$. The variation of *T*_1_ and *T*_2_ is a linear function of temperature over the expected range of bore temperatures (16°C to 26°C), with a variation at 3 T of 2.7 %/°C and 1.6 %/°C for *T*_1_ and *T*_2_, respectively.

### 2.4 Resolution and slice profile insets

**FIGURE 4:**
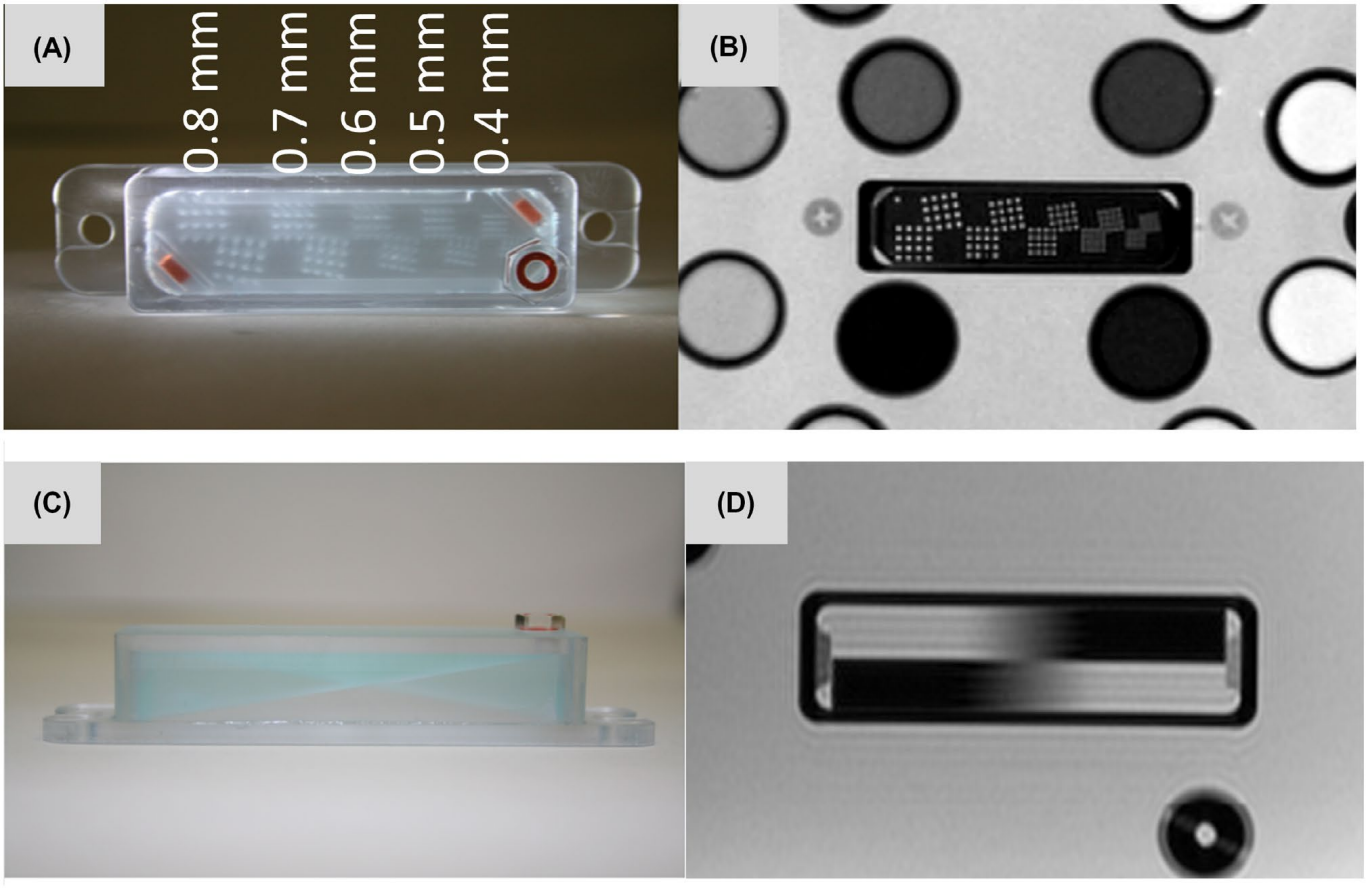
A,B, Photo and MRI of resolution inset. C,D, Photo and MRI of slice profile inset.

The resolution insets are modeled on the ACR guidance,^13^ and the slice profile wedges are designed in accordance with National Electrical Manufacturers Association (NEMA) MS 5‐2018 standard.^14^ Photographs and MR images of resolution and slice profile insets are shown in Figure 4.

The phantom contains two resolution insets consisting of 4 × 4 arrays of holes. A coarse resolution inset is drilled directly into plate 4 with hole diameters of 0.6 mm, 0.7 mm, 0.8 mm, 0.9 mm, and 1.0 mm ± 0.1 mm diameters. The fine resolution inset is contained within a rectangular prism filled with CuSO_4_ solution to give high contrast and has hole diameters of 0.4 mm, 0.5 mm, 0.6 mm, 0.7 mm, and 0.8 mm ± 0.1 mm diameters. Each hole array is duplicated at a 10° angle, sharing one common hole, to assess resolution along non‐principal axes. In total, there are 155 holes in each of the resolution insets (5 hole sizes, 31 per size). The advantage of this resolution inset is that it is easy to interpret visually, compact, easy to manufacture, and there is considerable community experience with it.

The slice profile inset consists of two 10° angle wedges contained within a rectangular prism filled with CuSO_4_ solution. The wedges are oriented so that one has a positive and one has a negative inclination. The presence of a pair of wedges allows for the slice profile to be corrected for the misalignment of the slice plane relative to the base plane of the wedges.

### 2.5 Safety

For most of the imaging studies, the shell was filled with 4 L of deionized water. This was done for two reasons: (1) safety, if the phantom spilled, the outer fill has no toxicity or disposal issues; and (2) practicality, the phantom could be easily refilled at any site. The main toxicity issue is due to the NiCl_2_ solutions. A total of 52 mg Ni^2+^ is included in the phantom. The NiCl_2_ is contained in the thick‐walled polypropylene contrast spheres that are double sealed with both a weld and epoxy. These spheres are very robust and can take large impacts without breakage or leakage.

### 2.6 SI traceability

A calibration object requires its properties to be precisely measured and traceable to a common set of definitions and units. The measured values must include a well‐defined uncertainty. A protocol for traceable spin relaxation measurements for MRI phantoms can be found in NIST SP250‐97^15^ and measurement detail can be found in Supporting Information Sec. 6. Spin relaxation time measurements are SI traceable via a metrology NMR system with a calibrated time base. The NMR time base, a 10 MHz oven‐controlled crystal oscillator, is calibrated against the NIST hydrogen maser clock. Time base errors, which include jitter of transmit and receive events, give minor contributions to the overall uncertainty in relaxation time measurements.

More important factors that determine the uncertainty in relaxation time measurements are RF transmit field $B_{1}^{+}$ calibration, $B_{1}^{+}$ inhomogeneity, *B*_0_ inhomogeneity, and measurement temperature. $B_{1}^{+}$ is calibrated using a nutation experiment on the sample to be calibrated, where the spins are progressively tipped, by varying the RF pulse duration, at increasing angles through at least 2 cycles (720°). Free induction decays are recorded, Fourier transformed to obtain a spectral peak, integrated and plotted against RF pulse duration. The data, which approximate a damped sinusoid, are fit to extract the pulse duration required for the desired tip angle and to extract an RF field inhomogeneity in the system. The RF field inhomogeneity is minimized during calibration by precisely centering a 10 mm long test sample within a 14 mm long RF homogenous zone. This field inhomogeneity and uncertainty in the $B_{1}^{+}$ calibration are fed into Monte Carlo Bloch simulations, along with all other uncertainties, to calculate the uncertainties in the measured relaxation times.

*B*_0_ inhomogeneity in the NMR metrology system is minimized by performing an automated shimming procedure on the sample to be calibrated. The inhomogeneous line width is required to be no more than 10 Hz larger than the homogenous line width. Monte Carlo Bloch simulations^15^ indicate that this level of *B*_0_ inhomogeneity gives a negligible contribution to the overall uncertainty in relaxation times with the pulse sequences used (inversion recovery sequence with composite 180° pulses for *T*_1_ and Carr‐Purcell‐Meiboom‐Gill (CPMG) sequence with 2.0 ms refocusing times for *T*_2_).

Temperature measurement and control are a major source of uncertainty both in the primary calibrations and in phantom‐based MRI scanner assessment. Phantom components can have several percent change in relaxation time per °C, Supporting Information Sec. 9. For primary calibration in the NMR metrology system, sample capillaries are immersed in a fluorinated heat transfer solution within a 5 mm diameter NMR tube. The fluorinated solution also serves as a susceptibility matching solution. A fiber optic thermometer is positioned 15 mm away from the sample and is calibrated in the same NMR tube in a water bath against two NIST‐calibrated platinum resistance thermometers.

Geometric accuracy is determined at manufacture time using computer‐controlled machining techniques. Post‐assembly geometric accuracy can be checked with computed tomography techniques using SI traceable geometric calibrations. Here, the geometric distortion is reported relative to the 3D model values, with uncertainty given by the manufacturing tolerances.

Composition of the MR parameter arrays is verified using inductively coupled plasma‐ mass or optical spectroscopy techniques calibrated with traceable NIST reference standards. A reference library of all solutions, flame sealed in capillaries, is kept to establish stability over time. A test of the stability of proton relaxation times of an archived sample of Ni‐12 solution at 3 T and 20.0°C, for example, gave *T*_1_ = 44.53 ms ± 0.01 ms, *T*_2_ = 31.86 ms ± 0.03 ms in May 2015 and *T*_1_ = 44.67 ms ± 0.003 ms, *T*_2_ = 31.97 ms ± 0.026 ms in June 2019, a difference of 0.3 % for *T*_1_ and 0.3 % for *T*_2_. Here, the reported errors are the SD of three consecutive measurements at each time point and is a measure of system stability and is not the uncertainty in the values, which must incorporate all systematic errors as described in NIST SP250‐97. Uncertainties will depend on the material under test, the precise pulse sequence used, the geometry of the sample, and on the values of the relaxation times. The uncertainty for proton spin relaxation time calibrations, defined as the range within which there is greater than 96 % probability of the true value, are given in the NIST calibration certificates for each material and are on the order of 1.5 %.

## 3 RESULTS

### 3.1 Geometric distortion, image uniformity, and B_1_ homogeneity

Geometric distortion is assessed in a manner similar to that described in Ref. 4. A 3D gradient echo image, with short echo time (TE), of the whole phantom is obtained with isotropic voxels with typical voxel dimension of 1.0 mm. This sequence can be used to assess the intrinsic geometric accuracy of the scanner, whereas sequences with longer TEs will be more sensitive to distortions due to the patient/phantom. The apparent locations of the 10.0 mm fiducial spheres are determined by cross‐correlation maximums between a fiducial sphere mask and the image (discussed in the Supporting Information). The sphere center can be determined to <0.1 mm, which is comparable to the accuracy in the construction. Determination of the center of the fiducial spheres is sensitive to defects, such as small air bubbles in the solution. These defects can be detected manually or numerically, and the defective sphere data discarded. After locating the apparent sphere centers, a similarity transformation (an affine transformation consisting of a translation, rotation, and isotropic scale factor), which minimizes the distances between the prescribed and apparent locations is determined by a generalized Procrustes algorithm. The resulting rotation and translation define the map from phantom to scanner coordinates. The scale factor provides a measure of the overall dimensional accuracy of the scanner. We define geometric distortion as the difference between the apparent position ${\vec{R}}_{a}$ and the prescribed position ${\vec{R}}_{p}$ of the sphere centers after the similarity transformation has been applied to the latter, $\delta \vec{R}={\vec{R}}_{a}‐{\vec{R}}_{p}$. An example geometric distortion analysis for an image without corrections for gradient non‐uniformity, is shown in Figure 2. A large (~2 mm) *x*‐component of geometric distortion is seen on the right face, with a negative sign, in Figure 2 since the apparent position is farther along the negative *x*‐axis than the prescribed position. Similarly, there is a large *x*‐component of geometric distortion on the left face, with a positive sign, since the apparent position is farther along the positive x‐axis. Examples of geometric distortion with non‐uniform gradient corrections applied are given in the Supporting Information and show the need for the phantom geometric accuracy to be < 0.1 mm. Local volumetric distortion can be assessed by comparing cross correlation volumes.

We assess the uniformity of image intensity as part of this same analyses by calculating the integrated intensity of the 57 fiducial spheres. Figure 2 shows image uniformity obtained from a head coil, which shows signal loss in the inferior or chin region due to low receive coil sensitivity. The image uniformity analysis can also be done as part of other protocols, such as the of *T*_1_, *T*_2_ protocols.

Finally, $B_{1}^{+}$ mapping can be done with the system phantom fiducial array using flip angle mapping techniques.^16^, ^17^, ^18^ The fiducial spheres, as well as the surrounding background fill liquid (DI water), provide regions with identical properties to allow identification of RF non‐uniformities. To assess the effect of fill conductivity and dielectric resonances, the fill solution can readily be swapped out with a conducting salt solution or a low dielectric constant fluorocarbon fluid. We do not perform this analysis here.

### 3.2 T_1_ measurement

**FIGURE 5:**
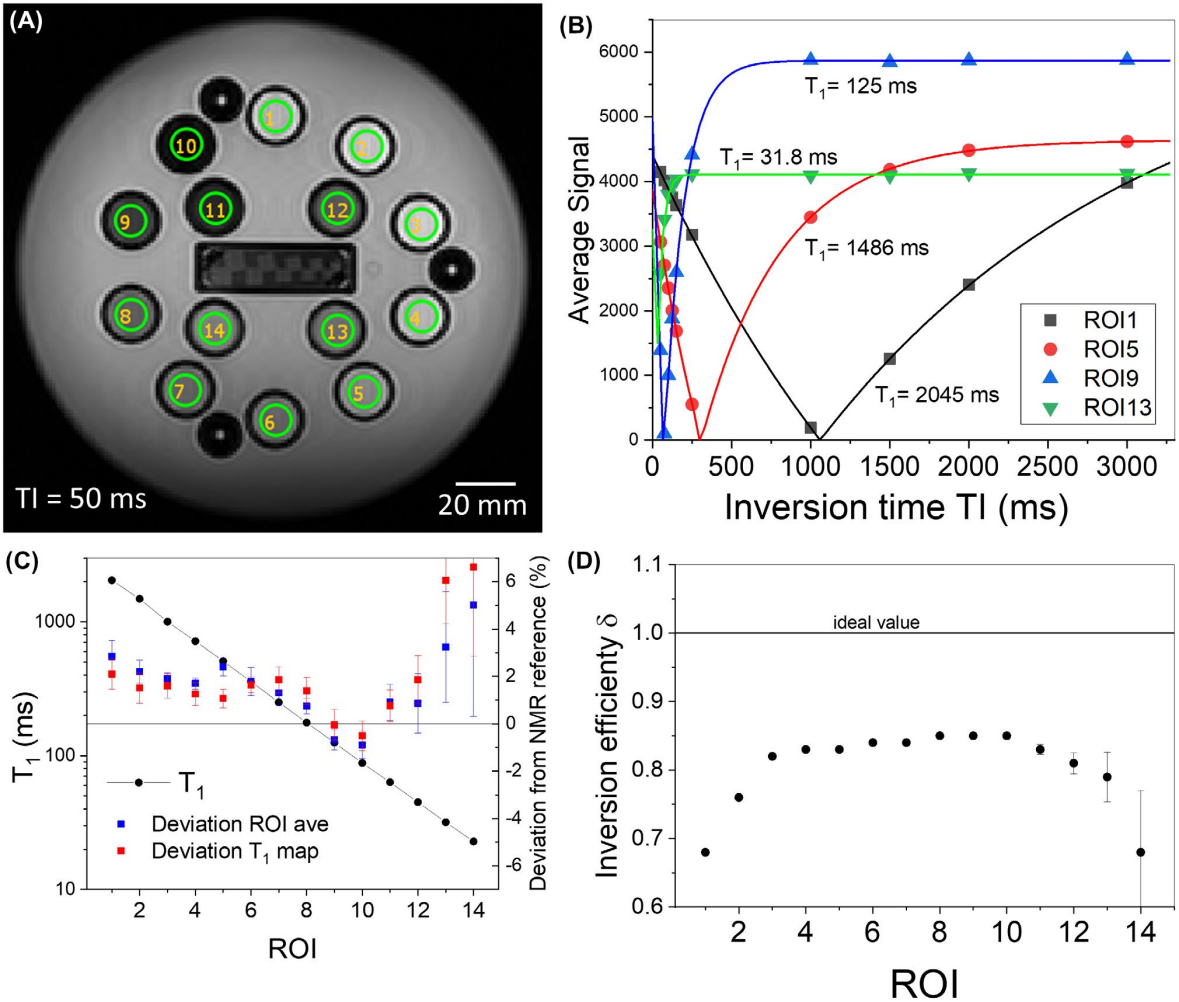
A, Coronal image of the NiCl_2_ array at 3 T ~20°C using an IR protocol. B, Average signal in ROIs 1, 5, 9, and 13 in the NiCl_2_ array, plotted as a function of TI and fits to the inversion recovery model. C, Automated analysis of *T*_1_ for each of the 14 NiCl_2_ array elements, with the deviation from the NMR reference measurements. The analysis was done both by (1) fitting the average of the ROI signal to the model (blue squares) and (2) fitting each voxel independently to the model, making a *T*_1_ map, and then averaging (red squares). The deviations are shown in blue and red squares, respectively. The error bars on the deviations are the standard errors in the nonlinear least squares fits and represent the quality of the model and the fit. D, The inversion efficiency, δ determined from the fits. The line at δ = 1 represents perfect inversion, and the error bars are the standard errors in the parameter determined from the nonlinear least squares fits. Unobserved error bars are contained within the symbol.

Figure 5 shows automated *T*_1_ analysis of the NiCl_2_ array using the Python‐based analysis package. Figure 5A shows an image of the NiCl_2_ array using the inversion‐recovery (IR) sequence protocol described in the Table S8 with recommended 10.0 mm circular ROIs centered on the NiCl_2_ array spheres. The suggested *T*_1_‐IR protocol requires 10 inversion times, TI, ranging from 50 ms to 3000 ms and TR = 4500 ms. Note, the actual inversion recovery protocol may vary slightly among the different vendors due to details of the scanner design and programmable control. Figure 5B shows a typical fit to the data using a standard IR model,^19^, ^20^, ^21^ appropriate when TR >> *T*_1_, and the inversion and detection flip angles are precisely 180° and 90°. The average signal in each ROI is given by $S\left(\mathrm{TI}\right)=A\left|1‐\left(1+\delta \right)e^{‐\frac{\mathrm{TI}}{T_{1}}}\right|$, where A is the signal amplitude when the magnetization is allowed to fully relax back to its equilibrium state, and δ is the inversion efficiency,^19^, ^21^, ^22^ the signal amplitude immediately following inversion normalized to A. Here, only magnitude data are used since the phase data are often not available and can be hard to interpret. This is in contrast with the NMR IR reference protocol, which uses the real part of the complex signal for analysis. Figure 5C shows the results of the *T*_1_ analysis along with the deviation relative to the NMR reference values given in Table S1. The deviation is defined as 100(*T*_1m_ − *T*_1r_)/*T*_1r_, where *T*_1m_, *T*_1r_ are the measured and reference values, respectively. The large deviation for sphere Ni‐14, which has the shortest *T*_1_, results from having only three data points where the signal is changing before full recovery. This is reflected in the large error bar, which is the standard error in the non‐linear least squares fit. The analysis was done both by (1) fitting the average of the ROI signal to the model and (2) fitting each voxel independently to the model, making a *T*_1_ map, and then averaging. Both methods agree within the standard errors. A series of *T*_1_ measurements made on 3 T scanners over the course of 7 years is shown in the Figure S13 to illustrate phantom stability.

More information is obtained from these fits than simply the relaxation time. The inversion efficiency, δ, is an important indicator of *B*_1_ and flip angle uniformity. As seen in Figure 5D, the inversion efficiency is close to 0.85, whereas the ideal value is 1.0. Typical values obtained from NMR data on the same solutions, with a more homogenous RF field geometry and using composite inversion pulses, are between 0.97 and 1.0. A Monte Carlo study of Bloch simulations indicate that there is a correlation between the deviation of the inversion efficiency from its ideal value and the error in the *T*_1_ measurements.^15^

**FIGURE 6:**
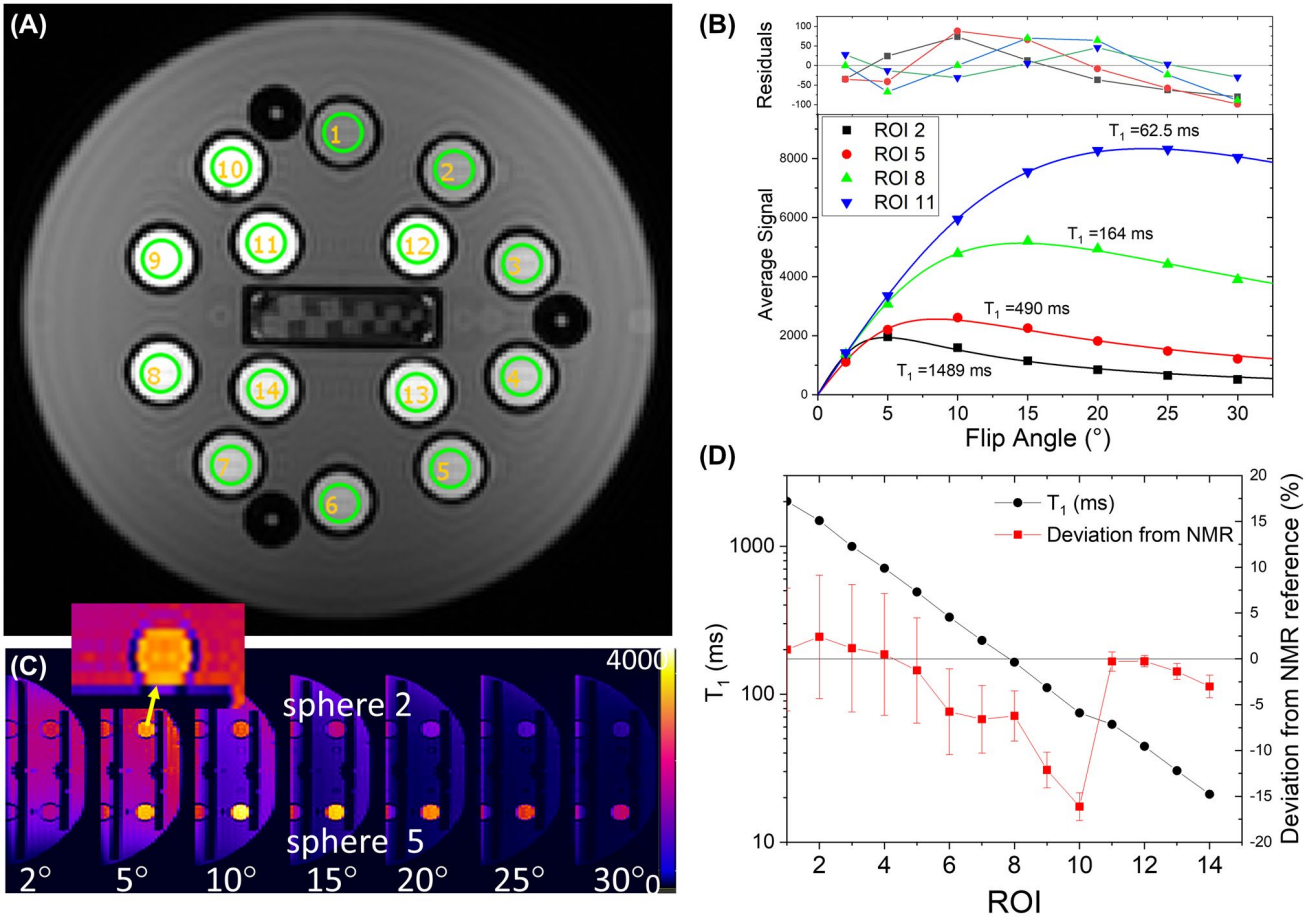
A, Coronal image of the NiCl_2_ array at 3 T ~20°C using a 3D gradient echo VFA sequence, B, Average signal and fit to VFA model for ROIs 2, 5, 8, 11. C, Sagittal images showing sphere 2 and sphere 5 for different flip angles. ROI 5 has a maximum signal near 10°, while ROI 2 has a maximum signal near 5°. The inset shows slice to slice variations that affect *T*_1_ values. D, Measured *T*_1_ and deviation from the NMR reference values for each of the 14 ROIs.

IR sequences, while fairly accurate in measuring *T*_1_, require long acquisition times. Variable flip angle (VFA) sequences, with shorter TRs and smaller flip angles, are considerably faster with less RF deposition. VFA protocols typically use 3D gradient echo sequences (see Supporting Information) and vary the flip angle α. Ideally, the signal is given by $S\left(\alpha \right)=S_{90}\sin\left(\alpha \right)\frac{1‐e^{‐\frac{\mathrm{TR}}{T_{1}}}}{1‐e^{‐\frac{\mathrm{TR}}{T_{1}}}\cos\left(\alpha \right)}$, where $S_{90}$ is the maximum signal given $\alpha =90^{\circ }$ and long TR.^20^, ^21^ Figure 6A shows a coronal image of the NiCl_2_ array using the VFA protocol described in the Supporting Information, with TR = 5.37 ms and TE = 1.49 ms. Figure 6C shows a series of sagittal images for a series of flip angles varying from 2° to 30°. The signal in each ROI has a maximum at the Ernst angle, $\cos\left(\alpha_{E}\right)=e^{‐\frac{\mathrm{TR}}{T_{1}}}$, which for ROI 5 is near 10° and ROI 2 is near 5°. Figure 6B shows the mean signal for ROIs 2, 5, 8, 11 along with fits to the model listed above and the residuals. As seen by the non‐Gaussian distribution of the residuals, there are systematic deviations from the simple model listed above due, in large part, to the inability to achieve the prescribed flip angle at each location in the phantom. Figure 6D shows the measured *T*_1_ values and deviations from the NMR reference values. The error bars in Figure 6D are the standard error in the fits, which are large for the longer *T*_1_ ROIs. The large standard errors on the fits, along with a systematic variation in the residuals, indicate that the model, which assumes an accurately prescribed flip angle that is the same for all regions in the phantom, is limiting measurement accuracy. More advanced models and protocols exist to improve on VFA *T*_1_ measurements^23^; however, here we are focusing on using the system phantom and simple protocols to identify and characterize scanner non‐idealities.

### 3.3 T_2_ measurement

**FIGURE 7:**
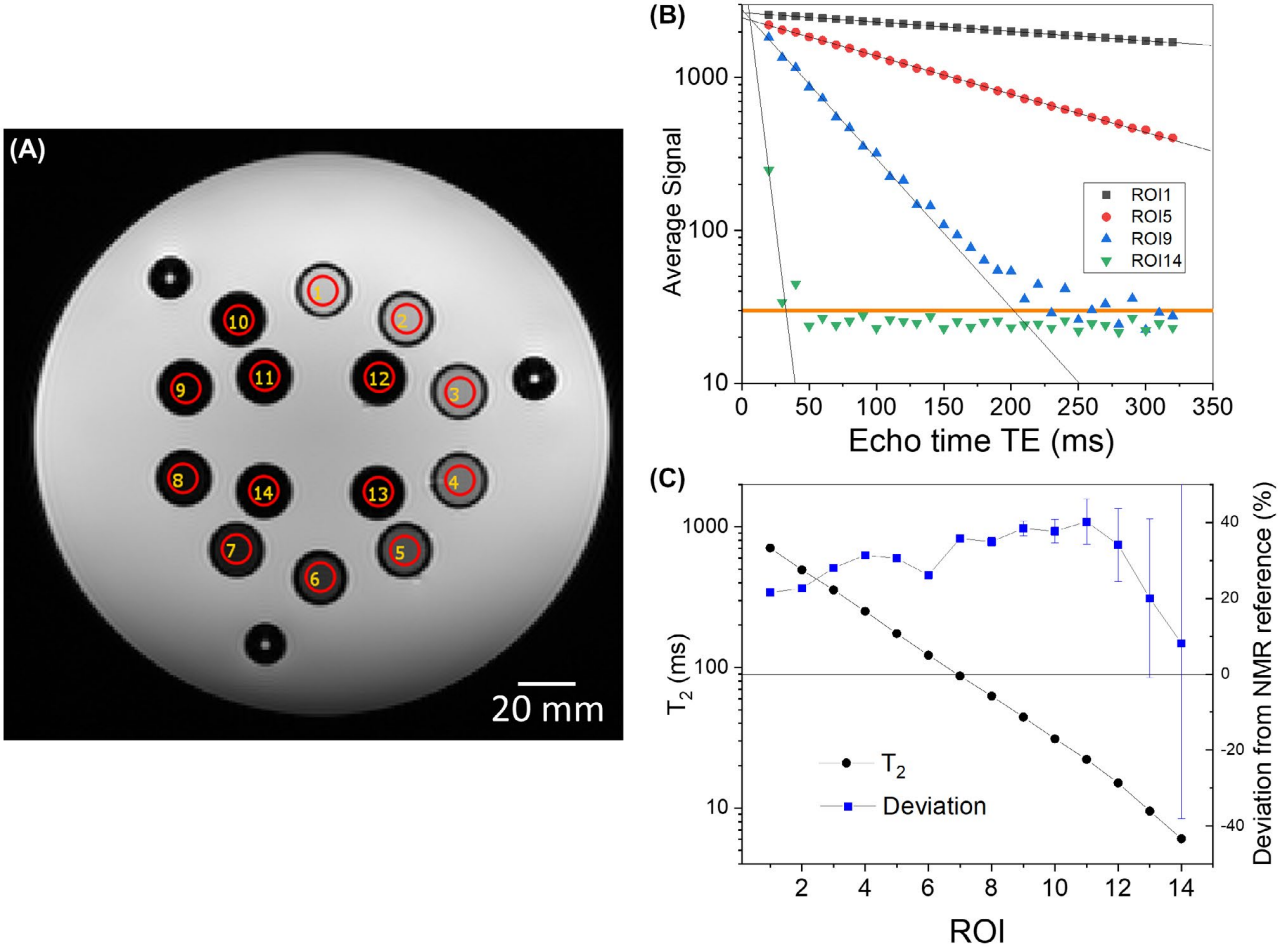
A, Coronal image of the MnCl_2_ array at 3 T ~20°C using a spin‐echo sequence with TR = 5000 ms. Typical fit to a spin echo protocol (B) and automated analysis of *T*_2_ (C) for each of the 14 array elements, with the deviation from the NMR reference measurements. The TE = 10 ms image was not used due to anomalously low signal, and points below the noise floor, the orange line in B, were not included in the fits.

Figure 7 shows *T*_2_ measurements of the MnCl_2_ array using the Python‐based analysis package. Figure 7A shows an image of the MnCl_2_ array using the spin echo (SE) sequence listed in the Supporting Information with TE varying from 10 ms to 320 ms by 10 ms and TR = 5000 ms. Figure 7B shows a typical fit to the data using a simple exponential model: $S\left(TE\right)=S_{0}e^{‐TE/T_{2}}$, where $S\left(\mathrm{TE}\right)$ is average signal in each ROI and TE is the spin TE. Figure 7C shows the *T*_2_ obtained from the fits along with the deviation relative to the NMR reference values given in Table S2. The error bars in Figure 7C are the standard errors derived from the non‐linear least squares fitting procedure. For the ROIs with short *T*_2_ times, there are not many points used in the fit, and the standard error is large.

### 3.4 Proton density and SNR measurement

**FIGURE 8:**
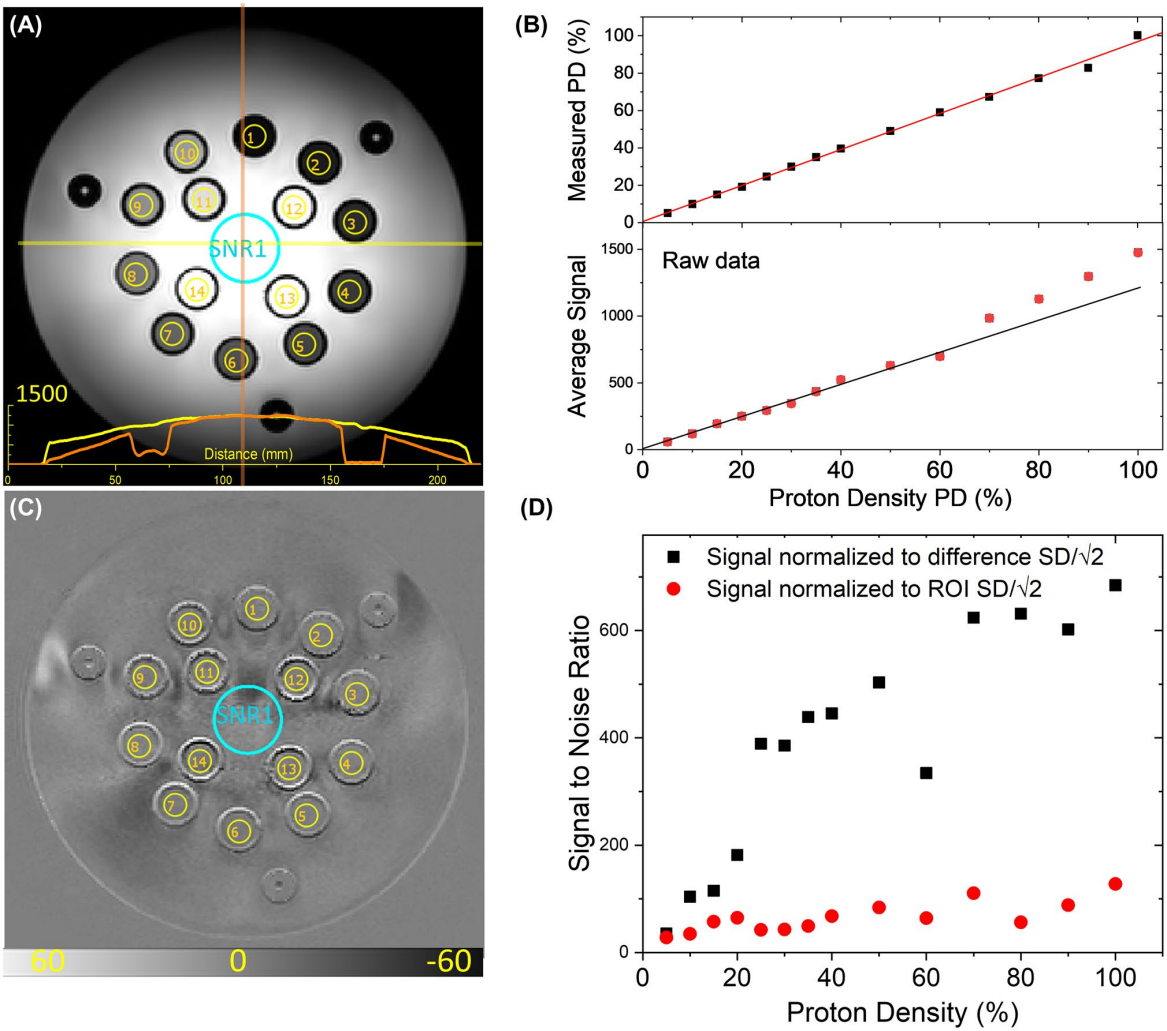
A, Coronal image of proton density array using a spin‐echo sequence with TR = 5000 ms, TE = 10 ms. A background SNR ROI is shown in blue, in addition to the 14 yellow proton density ROIs. Line scans across the center background fill are shown in yellow and orange at the bottom to show the image inhomogeneity. B, Average intensity of PD ROIs and measured PD as a function of prescribed proton density. C, Difference of two identical images and D, plot of the SNR as a function of proton density. Also shown in D, is the ratio of the signal to the SD within each ROI.

A coronal spin echo image of the PD array is shown in Figure 8A with the PD ROIs shown in yellow. Substantial background nonuniformity exists; the signal changes by a factor of ~6 going from the periphery to the center along the superior/inferior direction. Figure 8B shows the average signal in each PD ROI, and the measured MR proton density plotted versus prescribed proton density. The MR proton density is calculated by normalizing the average signal to the local background, as determined by averaging the signal from four points taken 3 mm outside the spheres, and then normalizing to the 100 % water sphere. The average signal in the PD ROIs shows a clear nonlinearity due to signal loss towards the periphery of the phantom. The normalized signal shows the expected linearity of the signal with respect to proton density. The maximum error in the measured proton density is 8.3 %, and the SD of the errors is 2.8 %.

In addition to measuring proton density, this array is used to measure SNR using methods similar to those described in Ref. 24. The noise is measured by taking the difference of two identical scans, $\Delta S=S_{2}‐S_{1}$, taken sequentially (Figure 8C), as discussed in Method 1 in Ref. 24. The SNR is calculated for each PD ROI by taking the average signal $S_{\mathrm{av}}=\frac{1}{2}\left(S_{2av}+S_{1av}\right)$ and dividing by the noise $N=SD/\sqrt{2}$, where SD is the SD of the difference signal ΔS within the ROIs. The SNR is plotted versus proton density in Figure 8D. Also shown is the average signal in each ROI normalized to the SD within each ROI. This is a measure of the ratio of the average signal to the nonuniformity within each region, only part of which comes from noise.

SNR1 ROI, shown in Figure 8 in blue, is meant to more closely mimic the geometry suggested in Ref. 24. However, the larger ROIs are more susceptible to scanner and phantom temporal drift, which gives rise to the low spatial frequency structure in Figure 8C.

### 3.5 Resolution

**FIGURE 9:**
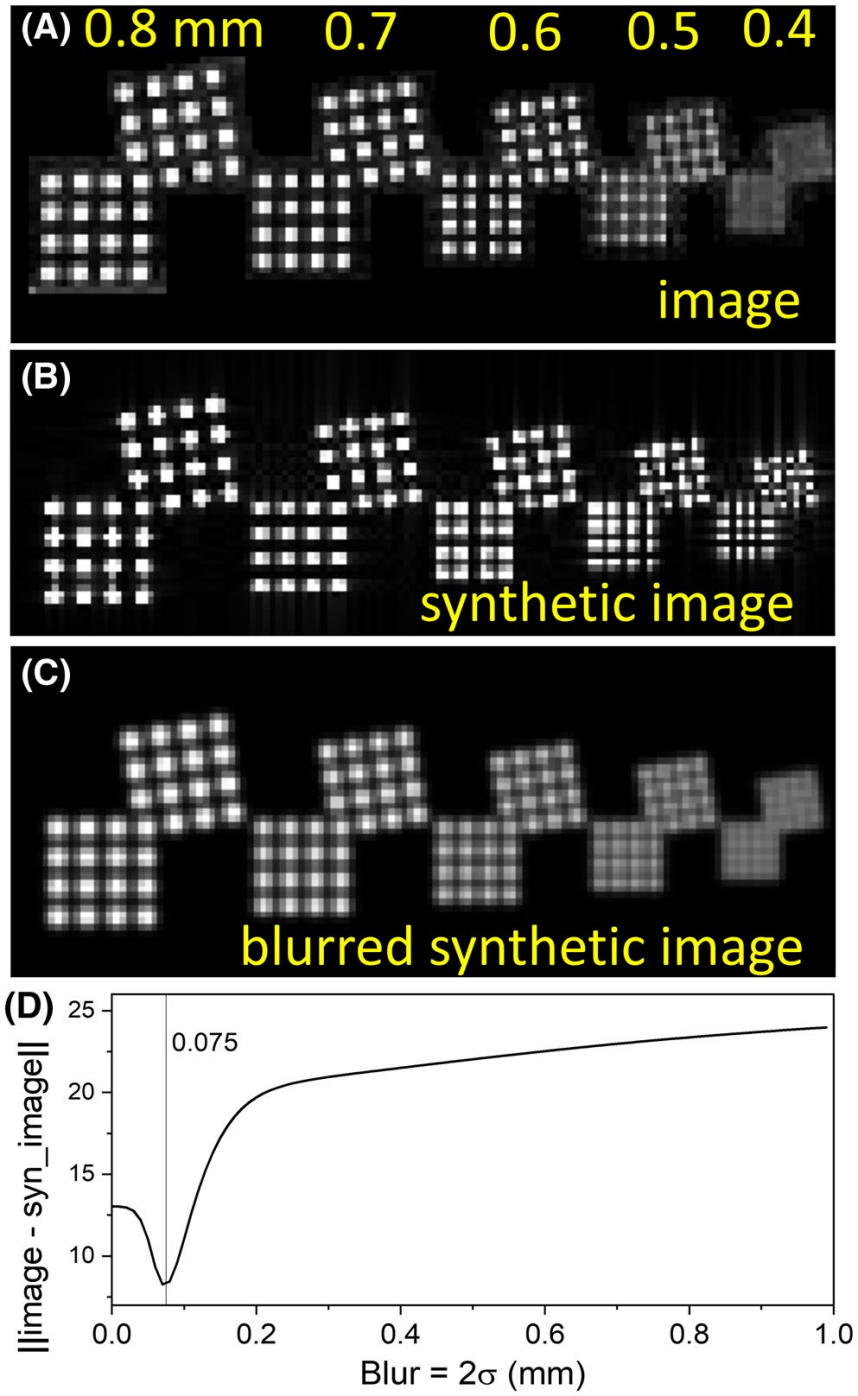
A, MRI of resolution inset taken with a voxel size of 0.35 mm and slice thickness of 4.0 mm. Here the resolution, according to ACR protocol is 0.5 mm since all the arrays with hole diameters ≥0.5 mm can be resolved, while the 0.4 mm hole size arrays cannot. B, Synthetic image of hole array with broadening only from finite k‐space sampling. C, Synthetic image with additional blurring from a Gaussian point spread function of width 2σ = 0.075 mm. D, The magnitude of the difference of the measured and synthetic image, using an L2 norm, as a function of the width of the point spread function, showing a minimum in the difference at a blurring of 0.075 mm.

Resolution is determined either using the manual method suggested by ACR^13^ or an automated computer algorithm that compares the MR image to synthesized images with different resolution distortions. Figure 9A shows an image of the resolution inset taken with a voxel size of 0.35 mm and a slice thickness of 4.0 mm. As per ACR guidelines, visual inspection of the image yields a resolution of 0.5 mm. Figure 9B shows a synthetic image generated by analytically computing the Fourier transform of the resolution‐inset disk array, calculating a finite k‐space image, and converting into a real‐space image. Comparison of the synthetic image to the measured image shows additional blurring beyond that expected from the point spread function due to finite k‐space sampling. Applying an additional Gaussian point spread function can improve the match between the synthetic and real image (Figure 9), with the difference between the images minimized using a point spread function with a width 2σ = 0.075 mm, as seen in Figure 9D. The resolution is then reported as a finite k‐space sample limited resolution of 0.35 mm and a machine‐specific Gaussian broadening of 0.075 mm.

### 3.6 Slice profile measurement

**FIGURE 10:**
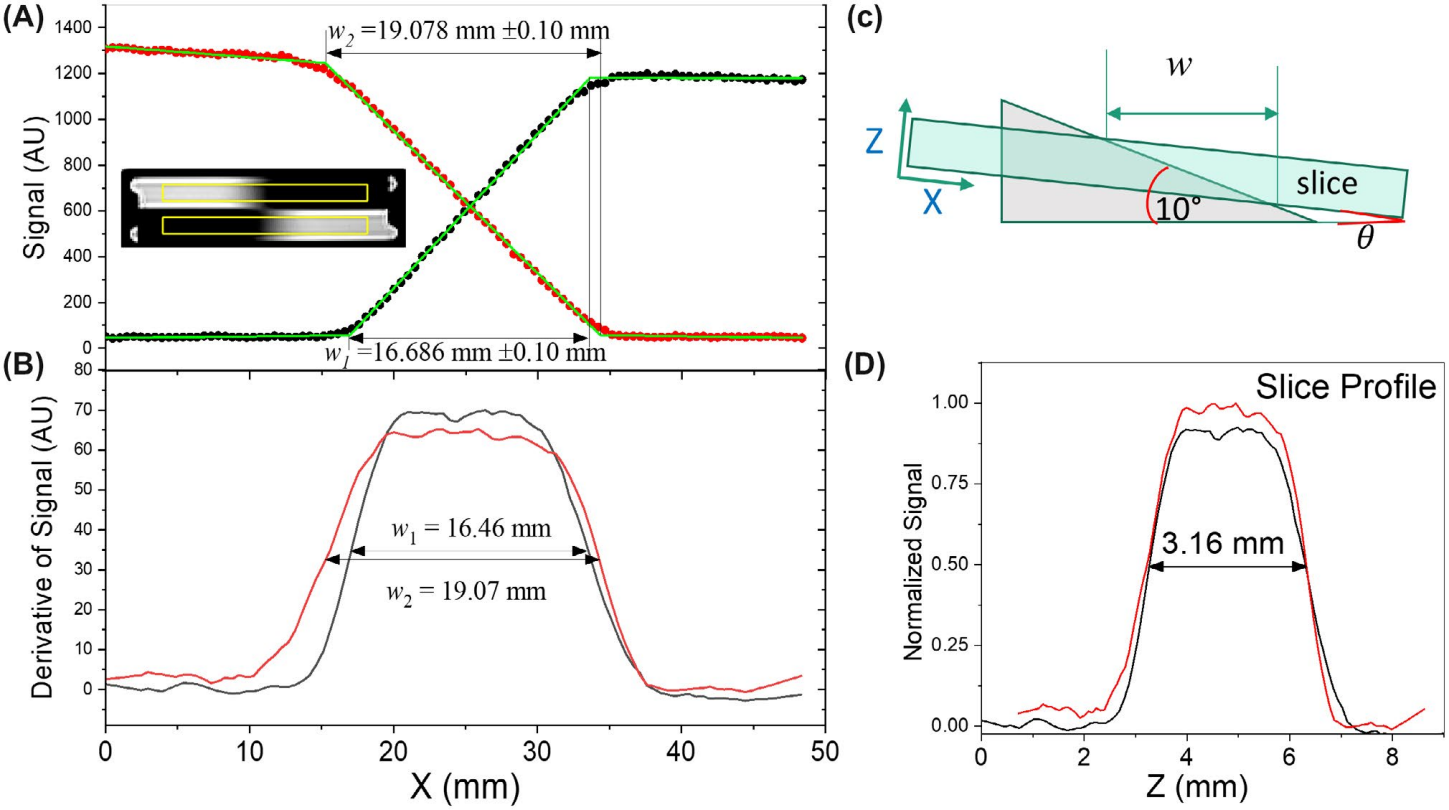
Slice profile analysis from an MR image with prescribed 3.0 mm slice thickness. A,B, The MR signal and its derivative, for rectangular ROIs centered on the two wedges (inset in A). C,D, The measurement geometry and the slice profile, corrected for angular misalignment *θ*. The measured slice thickness is 3.15 mm using the NEMA MS 5‐2018 protocol and 3.16 mm ± 0.2 mm using the automated protocol described in the text. The misalignment of the slice profile inset and the scan plane is *θ* = 0.72°.

Slice profile measurements, shown in Figure 10, are done in accordance with the NEMA MS‐5‐2018 standard^14^: the edge response function is calculated from the imaging data by placing a 4 mm x 50 mm rectangular ROI over each wedge, differentiating the data with respect to image position, and then multiplying the image position by tan(*θ*) to determine the slice profile. $\alpha =10^{\circ }+\theta$ is the corrected angle, where $\theta =1/2\sin^{‐1}\left(\left(w_{2}‐w_{1}\right)\sin\left(2*10^{\circ }\right)/\left(w_{2}+w_{1}\right)\right)$ measures the angle of the image plane with respect to the base of the wedges (see Figure 10C inset), and $w_{1},w_{2}$ are the projected thicknesses of the two wedges. The slice thickness, $t_{\mathrm{sl}},$ is then determined by measuring the full width at half maximum of the corrected slice profile. The suggested prescribed slice thicknesses are 3 mm and 5 mm, with the maximum being set by the wedge height of 10 mm.

The slice thickness may also be measured by fitting the edge response function with a piecewise linear function, extracting the projected slice thicknesses, and then multiplying by the tangent of the wedge angle: $t_{\mathrm{sl}}=\frac{1}{2}\left(w_{2}+w_{1}\right)\times \tan\left(10^{\circ }\right)$. Averaging both ramp orientations corrects, in lowest order, for errors due to the misorientation of the image slice plane relative to the base of the wedges. The latter method has the advantage that it is easily automated and has a well‐defined uncertainty.

## 4 DISCUSSION

The system phantom described here, or modified versions of it, have been used for several studies including repeatability of MR fingerprinting,^25^ assessing changes occurring during scanner upgrades,^26^ and assessing variation in multi‐site *T*_1_ measurements.^27^ Many other uses are ongoing, including monitoring of scanners for quality assurance and homogenizing scan protocols across multi‐vendor scanners. The phantom was designed to be used in several modes, starting from simple visual inspection for identification of geometric distortion and resolution, to semi‐automated analysis to determine relaxation times and SNR, and finally for custom analysis to validate advanced quantitative protocols. While application‐specific phantoms may provide better structures for particular measurements, for example, the ADNI phantom for geometric distortion or a uniform agar phantom for image homogeneity, the system phantom is meant for characterization of a large number of scanner parameters and to have sufficient accuracy, generality, and complexity to assess the accuracy of many types of advanced hardware and protocols. The standard protocols provided with the system phantom are not intended to be the best protocols for accurate measurement of geometric distortion, proton spin relaxation times, resolution, and SNR. Rather, they are meant as a common method to test the ideality of the scanner and to provide a comparison for other quantitative protocols that may be faster or more accurate.

Here we have shown how the phantom can be used for scanner diagnostics assessing geometric distortion, image homogeneity, resolution, and slice profile. The resulting data can provide both parameters to benchmark scanner performance and insight on what is determining these parameters. For example, resolution analysis can separate the pulse‐sequence component from the intrinsic scanner component of the point spread function. The SNR protocol can separate temporal and spatial noise components, which contribute in different ways to SNR. Finally, the MR‐parameter arrays provide a very broad range of properties to allow validation of both standard and advanced quantitative protocols.

One specific challenge encountered is that *T*_2_ is not a rigorously defined quantity.^28^ $1/T_{2}$ is the component of the total transverse spin dephasing rate, $1/T_{2}^{*}$, that is considered to be intrinsic to the sample, with the extrinsic component, $1/T_{2}^{\prime }$, being eliminated using a spin echo: $\frac{1}{T_{2}^{*}}=\frac{1}{T_{2}^{\prime }}+\frac{1}{T_{2}}$. For complex materials there is ambiguity on what dephasing components are considered extrinsic and intrinsic, and the components being eliminated by the refocusing pulses will depend on the details of the refocusing pulses. *T*_2_ can be a function of the refocusing time and the values measured by CPMG are not the same as measured by SE.^29^, ^30^ The system phantom uses simple materials, so this is not a major issue here. However, to get traceability, a robust operational definition of *T*_2_ is required. Here, we define it as the exponential signal decay measured with a CPMG sequence with a refocusing time of 2 ms. CPMG sequences also effectively null out the longitudinal magnetization, which, if present along with *B*_0_ and *B*_1_ inhomogeneities, can lead to stimulated echoes. Other imperfections such as imperfect refocusing pulses, which can be present in SE sequences, can add long‐time signal and can increase the measured *T*_2_. The variability in *T*_2_ measurements have been extensively studied in the literature.^31^, ^32^, ^33^ Here, we note that simple SE sequences, which may appear to be similar, can have very different spin manipulations and signal production. Using a standard phantom can identify these differences, which can be important for both conventional qualitative interpretation as well as quantitative analysis.

From the feedback of users of the prototype phantoms, several limitations and potential improvements have been identified. During the time since this phantom was conceived, head coil geometries are becoming more complex, and the size is decreasing. Newer head coils have minimum dimensions of 196 mm or less, which is smaller than the prototype system phantom design. There is a trade‐off in the system phantom between sampling large volumes to assess uniformity of measurements and the ability to fit into newer, smaller coils. Smaller phantom shells have been investigated and are available in commercial versions. Another concern is the cost of the phantom. Tradeoffs can be made, such as relaxing the 0.1 mm geometric accuracy, to reduce costs to make the phantoms commercially viable, as discussed in Supporting Information Sec. 13.

The contrast array components are arranged in an orderly fashion to allow easier interpretation by users. More information may potentially be obtained by random placement of the contrast elements, given that their properties are highly determined and errors due to location may be better extracted using computer algorithms. There could be benefit as well in reducing the number of unique spheres in the contrast array to provide replicate spheres. The prototype phantoms presented here are easily reconfigurable to test these modifications.

The contrast and fiducial arrays use plastic components that have sharp signal/‐to‐no‐signal interfaces that cause Gibb’s ringing artifacts, as can be seen in Figure 6A. These artifacts can confound SNR and other measurements. While these artifacts can be accounted for in certain measurements, for instance they can be included in the kernel for the cross‐correlations determining geometric distortion, it would be desirable to minimize these artifacts by making the plastic shells thinner or by allowing them to have some signal.

Dielectric resonances can give rise to image distortion, particularly for large water phantoms at high fields.^34^ These effects still need to be studied for the system phantom. However, the outer fill is easily changeable, without affecting the measurement protocols. Fills with different conductivities and dielectric properties can easily be explored.

Temperature control and monitoring remain a critical issue, especially when trying to verify the accuracy of relaxation time measurements to better than 2 %. While the system phantom is shipped with a thermometer to record temperature, the bore temperatures are often different from the ambient scanner room temperature or even the control room, where the temperature is typically measured. There is no guarantee that the phantom temperature is the same as the recorded temperature. Furthermore, the temperature information is not easily encoded in the DICOM files. This establishes the need for in‐situ NMR thermometers, and an MRI‐readable liquid crystal thermometer has been developed for MRI phantoms.^35^, ^36^ Furthermore, the system phantom would greatly benefit from the use of materials that are less sensitive to variations in temperature and magnetic field strength. Most paramagnetic salts such as MnCl_2_ and CuSO_4_ have considerable temperature^10^ and field dependence (see Supporting Information Figures S10 and S11) in their relaxivity values r_1_, r_2_ (mM^−1^ s^−1^). NiCl_2_, while having less temperature and field dependence, has safety/toxicity issues.

The materials used in the system phantom were chosen for their stability and simple properties, for example, exhibiting mono‐exponential spin relaxation. As shown in the Supporting Information, a reference library sample, sealed in a glass capillary, has 4‐year *T*_1_ and *T*_2_ stability better than 0.1 %. Assessing stability of the phantom components is more challenging, since sampling the solutions for SI‐traceable NMR measurements would be destructive. The plastic spheres in the phantoms have some permeability to water and may slowly absorb paramagnetic salts, leading to long‐term drift in *T*_1_ and *T*_2_. A sampling protocol of the MR‐property spheres, stored under conditions similar to those contained within the phantom, is required to assess long term stability and is in progress.

There is demand for more biomimetic materials that match multiple tissue properties, such as the apparent water diffusion coefficient as well as proton density and proton spin relaxation times. It is challenging to obtain complex biomimetic materials that are stable and also have properties that can be rigorously quantified. However, this work is underway, as illustrated by the stable fat mimic developed for the commercial version of the breast phantom developed by NIST and the University of California San Francisco.^37^

While the current implementation of the MRI system phantom can be improved and will never be suitable for all calibration needs, it is a first attempt at developing and implementing a rigorous calibration structure with traceable properties, community‐driven open construction details and evaluation protocols, and long‐term monitoring and assessment with state‐of‐the‐art metrology tools.

## CONFLICT OF INTEREST

Todor Karaulanov is an employee of CaliberMRI.

## Supporting information


**FIGURE S1** A, The hemispherically machined surface on the post matches with the inclined notches, B, to precisely locate the plates. Photos of blue fiducial, C, and red MnCl_2_ array, D, spheres showing the sealing and mounting methods. D, inset shows micro‐computed tomography image of contrast sphere
**FIGURE S2** Relaxation rates as a function of paramagnetic salt concentration measured by inductively coupled Plasma (ICP) mass spectrometry at 1.5 T and 3.0 T. The solid lines are fits assuming a linear increase in relaxation rate with concentration and a zero‐concentration intercept given by the measured values for high purity water
**FIGURE S3** Schematic of the fiducial array analysis showing input 3D image of the system phantom, cropped image with just the fiducial spheres, image of fiducial sphere, synthetic k‐space image and real‐space image used as a convolution mask, slice of 3D convolution image, and convolution profiles with fits used to obtain sphere center
**FIGURE S4** Fiducial analysis on a 1.5 T scanner with a gradient echo sequence. A, Coronal slice, B, axial slice, C, sagittal slice. D, magnified image of fiducial sphere image with ROI location after automated location. E, Normalized integrated intensity for all 57 fiducial spheres. F, Difference between center of mass and convolutional sphere centers
**FIGURE S5** A, B, C, Geometric distortion of the 57 fiducial sphere centers in x, y and z directions, respectively
**FIGURE S6** NMR signal for Ni‐12 versus inversion time for the T1‐IR protocol along with fits to the model described in the text. Data for three consecutive measurements are shown along with the residuals for each measurement (top plot). The errors listed for *T*_1_ and the inversion efficiency are the standard deviation of the 3 values obtained for each of the measurements
**FIGURE S7** NMR signal from Ni‐12 using a CPMG sequence as a function of acquisition time along with exponential fits. Data for three consecutive measurements are shown along with the residuals for each measurement (top plot). The errors listed for *T*_2_ are the standard deviation of the 3 values obtained for each of the measurements
**FIGURE S8** Magnetic field dependence of *T*_1_ and *T*_2_ for the MnCl_2_ and NiCl_2_ arrays
**FIGURE S9** Temperature dependence of *T*_1_, *T*_2_ for the Ni‐12 solution measured in a metrology NMR at 3.0 T. The plot shows data from a flame‐sealed borosilicate‐capillary library sample over the course of 4 years
**FIGURE S10** Variation of normalized relaxation times with temperature for the NiCl_2_ array at 3.0 T
**FIGURE S11** Temperature coefficient of spin relaxation times for the MnCl_2_ array at 3.0 T
**FIGURE S12** Variation of relaxation times with temperature for the CuSO_4_ fiducial solution at 3.0 T
**FIGURE S13** Deviation of T1‐IR values from NMR reference values at 3 T over the course of 7 years. The gray bar indicated the range of values given a phantom temperature that can vary between 18°C and 22°C
**FIGURE S14** Water mass uptake for various plastics: nylon/polyamide (PA), poly(methyl methacrylate) (PMMA), polycarbonate (PC), polyvinyl chloride (PVC), polyvinylidene fluoride (PVDF), polyphenylene sulfide (PPS), polypropylene (PP). The samples were 25 mm diameter, 6 mm thick disks
**FIGURE S15** Geometric distortion during water soaking of the same samples used in Figure S13. The horizontal line indicates the threshold for maintaining the specified geometric distortion of the phantom plates
**FIGURE S16** Spin relaxation times for NiCl_2_‐3, NiCl_2_‐5, NiCl_2_‐10 at 3 T, 20°C as a function of pH
**FIGURE S17** Relaxation times for ACS‐grade and deionized water as a function of temperature
**TABLE S1** NiCl_2_ Array
**TABLE S2** MnCl_2_ Array
**TABLE S3** Proton Density Array
**TABLE S4** Water proton spin relaxivities determined from the slope of the data in Figure S2
**TABLE S5** Isotropic Volume Series
**TABLE S6** Section Thickness Series
**TABLE S7** Resolution Inset Series
**TABLE S8** Proton Density and Signal to Noise Series
**TABLE S9** *T*_1_ Inversion Recovery Series
**TABLE S10** *T*_1_ Variable Flip Angle Series
**TABLE S11** *T*_2_ Series
**TABLE S12** Starting Chemicals for Contrast Fluids
**TABLE S13** NiCl_2_ Solutions
**TABLE S14** MnCl_2_ Solutions
**TABLE S15** Proton Density Solutions

## Data Availability

The analysis software can be found at https://github.com/MRIStandards/PhantomViewer. An analysis manual, 3D models, and sample datasets can be found at https://github.com/MRIStandards/SystemPhantom.
