# Supplementary material for: A standard system phantom for magnetic resonance imaging
Source: Magn Reson Med. 2021 Apr 13;86(3):1194–211. doi: 10.1002/mrm.28779 (PMC8252537; doi:10.1002/mrm.28779)
Supplement: Supplementary file 1 — FIGURE S1 A, The hemispherically machined surface on the post matches with the inclined notches, B, to precisely locate the plates. Photos of blue fiducial, C, and red MnCl2 array, D, spheres showing the sealing and mounting methods. D, inset shows micro‐computed tomography image of contrast sphere FIGURE S2 Relaxation rates as a function of paramagnetic salt concentration measured by inductively coupled Plasma (ICP) mass spectrometry at 1.5 T and 3.0 T. The solid lines are fits assuming a linear increase in relaxation rate with concentration and a zero‐concentration intercept given by the measured values for high purity water FIGURE S3 Schematic of the fiducial array analysis showing input 3D image of the system phantom, cropped image with just the fiducial spheres, image of fiducial sphere, synthetic k‐space image and real‐space image used as a convolution mask, slice of 3D convolution image, and convolution profiles with fits used to obtain sphere center FIGURE S4 Fiducial analysis on a 1.5 T scanner with a gradient echo sequence. A, Coronal slice, B, axial slice, C, sagittal slice. D, magnified image of fiducial sphere image with ROI location after automated location. E, Normalized integrated intensity for all 57 fiducial spheres. F, Difference between center of mass and convolutional sphere centers FIGURE S5 A, B, C, Geometric distortion of the 57 fiducial sphere centers in x, y and z directions, respectively FIGURE S6 NMR signal for Ni‐12 versus inversion time for the T1‐IR protocol along with fits to the model described in the text. Data for three consecutive measurements are shown along with the residuals for each measurement (top plot). The errors listed for T 1 and the inversion efficiency are the standard deviation of the 3 values obtained for each of the measurements FIGURE S7 NMR signal from Ni‐12 using a CPMG sequence as a function of acquisition time along with exponential fits. Data for three consecutive measurements are shown along with the residual [file MRM-86-1194-s001.pdf]

Supporting Information

A Standard System Phantom for Magnetic Resonance Imaging

20210323

Contents

1. Phantom Construction 3
2. MR Parameter Array Tables 4
3. Recommended Imaging Protocols 7
4. Materials Database 14
5. Fiducial Array/ Geometric Distortion Measurements 18
6. NMR Calibration Measurements 21
7. Field, Temperature, and Time Stability 24
8. Material Stability 29
9. The Commercial System Phantom 32

Supporting Information Figures

FIGURE S1 A, The hemispherically-machined surface on the post matches with the inclined notches, B, to precisely locate the plates. Photos of blue fiducial, C, and red $MnCl_2$ array, D, spheres showing the sealing and mounting methods. Insets show X-ray computed tomography images of the fiducial and MR-parameter spheres. The fiducial spheres are precisely machined while the molded contrast spheres show thicker non-spherical regions near the weld joint. 3

FIGURE S2 Relaxation rates as a function of paramagnetic salt concentration measured by inductively coupled Plasma (ICP) mass spectrometry at 1.5 T and 3.0 T. The solid lines are fits assuming a linear increase in relaxation rate with concentration and a zero-concentration intercept given by the measured values for high purity water. 6

FIGURE S3 Schematic of the fiducial array analysis showing input 3D image of the system phantom, cropped image with just the fiducial spheres, image of fiducial sphere, synthetic k-space image and real-space image used as a convolution mask, slice of 3D convolution image, and convolution profiles with fits used to obtain sphere center. 19

FIGURE S4 Fiducial analysis on a 1.5 T scanner with a gradient echo sequence. A, Coronal slice, B, axial slice, C, sagittal slice. D, magnified image of fiducial sphere image with ROI location after automated location. E, Normalized integrated intensity for all 57 fiducial spheres. F, Difference between center of mass and convolutional sphere centers. 19

FIGURE S5 A, B, C, Geometric distortion of the 57 fiducial sphere centers in *x*, *y* and *z* directions. 20

FIGURE S6 NMR signal for Ni-12 versus inversion time for the T1-IR protocol along with fits to the model described in the text. Data for three consecutive measurements at 3 T and 20 °C, are shown along with the residuals for each measurement (top plot). The errors listed for T_1 and the inversion efficiency are the standard deviation of the 3 values obtained for each of the measurements. 22

FIGURE S7 NMR signal from Ni-12 using a CPMG sequence as a function of acquisition time along with exponential fits. Data for three consecutive measurements at 3 T and 20 °C, are shown along with the residuals for each measurement (top plot). The errors listed for T_2 are the standard deviation of the 3 values obtained for each of the measurements. 23

FIGURE S8 Magnetic field dependence of T_1 and T_2 for the $MnCl_2$ and $NiCl_2$ arrays. 24

FIGURE S9 Temperature dependence of T_1, T_2 for the Ni-12 solution measured in a metrology NMR at 3.0 T. The plot shows data from a flame-sealed borosilicate-capillary library sample over the course of 4 years. 25
FIGURE S10 Normalized relaxation times versus temperature for the $NiCl_2$ array at 3.0 T. 26
FIGURE S11 Temperature coefficient of spin relaxation times for the $MnCl_2$ array at 3.0 T............................. 27
FIGURE S12 Relaxation times versus temperature for the $CuSO_4$ fiducial solution at 3.0 T. 27
FIGURE S13 Deviation of T_1-IR values from NMR reference values at 3 T over the course of 7 years. The gray bar indicates the expected range of values given a phantom temperature that can vary between 18 °C and 22 °C.. 28
FIGURE S14 Water mass uptake for various plastics: nylon/polyamide (PA), poly(methyl methacrylate) (PMMA), polycarbonate (PC), polyvinyl chloride (PVC), polyvinylidene fluoride (PVDF), polyphenylene sulfide (PPS), polypropylene (PP). The samples were 25 mm diameter, 6 mm thick disks........................ 29
FIGURE S15 Geometric distortion during water soaking of the same samples used in Figure S14. The horizontal line indicates the threshold for maintaining the specified geometric distortion of the phantom plates........ 30
FIGURE S16 Spin relaxation times for $NiCl_2$-3, $NiCl_2$-5, $NiCl_2$-10 at 3T, 20 °C as a function of pH................ 31
FIGURE S17 Relaxation times for ACS-grade and deionized water as a function of temperature..................... 31

Supporting Information tables

TABLE S1: $NiCl_2$ Array 4
TABLE S2: $MnCl_2$ Array... 5
TABLE S3: Proton Density Array 5
TABLE S4: Water proton spin relaxivities determined from the slope of the data in Figure S2........................... 6
TABLE S5: Isotropic Volume Series.. 7
TABLE S6: Section Thickness Series... 8
TABLE S7: Resolution Inset Series... 9
TABLE S8: Proton Density and Signal to Noise Series 10
TABLE S9: T_1 Inversion Recovery Series.. 11
TABLE S10: T_1 Variable Flip Angle Series 12
TABLE S11: T_2 Series 13
TABLE S12: Starting Chemicals for Contrast Fluids.. 14
TABLE S13: $NiCl_2$ Solutions 15
TABLE S14: $MnCl_2$ Solutions.. 16
TABLE S15: Proton Density Solutions 17

1. Phantom Construction

Figure S1 shows photos of the plate locating mechanism required to achieve 0.1 mm accuracy of fiducial sphere centers. The hemispherically-machined surface on the post, A, matches with the inclined notches, B, to precisely locate the plates. The plates and posts are machined from polyphenylene sulfide (PPS), which is annealed to relieve stresses before machining. PPS was chosen because of its high rigidity, precision machinability, and low water absorption. Photos of blue fiducial, and red $MnCl_2$, spheres are shown in Figure S1C, D along with X-ray computed tomography (CT) images.

The fiducial spheres, made from polyvinyl chloride (PVC), have a machined 10 mm internal diameter (ID) with a precise mounting and locating geometry. The base hemisphere and mounting stud are one machined piece, while the top hemisphere is glued into place, registered with a locating collar. PVC was chosen due to its good machinability, low water absorption, and easy joinability. PVC, due to its high density (1.38 g/cm^3) relative to water, shows high contrast in CT, which enables precise location of the fiducial spheres in CT images used in primary calibration of the geometric accuracy of the phantom.

The commercial polypropylene (PP) spheres used in the MR parameter arrays, do not have a precise spherical ID, as seen in CT image in the inset in D. Commercial PP spheres were chosen because they are inexpensive, very robust and difficult to fracture, and have the lowest water absorption of all materials tested. The robustness of the MR parameter spheres is an important safety component since some of these spheres contain Ni salts that are classified as toxic.

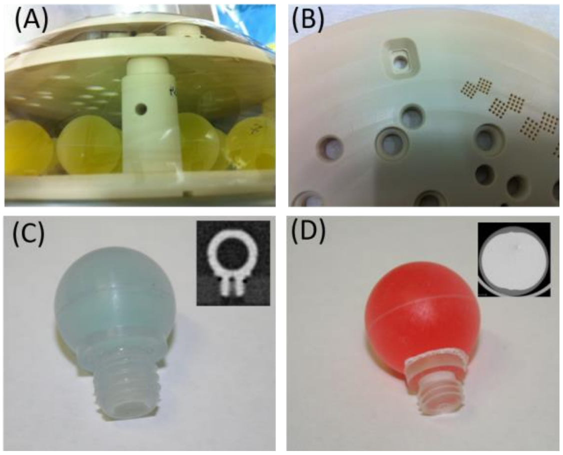

FIGURE S1 A, The hemispherically-machined surface on the post matches with the inclined notches, B, to precisely locate the plates. Photos of blue fiducial, C, and red $MnCl_2$ array, D, spheres showing the sealing and mounting methods. Insets show X-ray computed tomography images of the fiducial and MR-parameter spheres. The fiducial spheres are precisely machined while the molded contrast spheres show thicker non-spherical regions near the weld joint.

2. MR Parameter Array Tables

The following tables show properties of the magnetic resonance (MR) parameter arrays used in the prototype system phantoms at 20 °C. The $NiCl_2$ and $MnCl_2$ concentration are determined by inductively coupled plasma (ICP) techniques while the D_2O concentration is determined by gravimetric analysis. Figure S2 plots the relaxation rates $R_1 = 1/T_1, R_2 = 1/T_2$ for the $NiCl_2$ and $MnCl_2$ arrays and fits to a linear model

$$R_1 = r_1 C_1 + R_{1water}$$
$$R_2 = r_2 C_2 + R_{2water}$$

where R_{1water}, R_{2water} are the measured relaxation rates for high purity water. The relaxivities r_1, r_2 at 20 °C obtained from the fits are given in Table S4.

TABLE S1: $NiCl_2$ Array

Contrast ID	X (mm)	Y (mm)	Z (mm)	positional accuracy (mm)	ICP measured $NiCl_2$ Concentration (mM)	NMR Measured T_1 @ 1.5 T (ms)	T_1 Standard Deviation (ms)	NMR Measured T_2 @ 1.5 T (ms)	T_2 Standard Deviation (ms)	NMR Measured T_1 @ 3.0T (ms)	T_1 Standard Deviation (ms)	NMR Measured T_2 @ 3.0 T (ms)	T_2 Standard Deviation (ms)
Ni-1	0.0	56.5	50.0	±0.4	0.268	2 033	4.6	1 669	0.5	1 989	1.0	1 465	1.0
Ni -2	29.4	56.5	40.5	±0.4	0.556	1 489	1.4	1 244	0.6	1 454	2.5	1 077	1.8
Ni -3	47.6	56.5	15.5	±0.4	1.053	1 012	0.2	859.3	0.17	984.1	0.33	717.9	1.12
Ni -4	47.6	56.5	-15.5	±0.4	1.683	730.8	1.10	628.5	0.13	706	1.5	510.1	1.36
Ni -5	29.4	56.5	-40.5	±0.4	2.579	514.1	0.06	446.3	0.11	496.7	0.41	359.6	0.22
Ni -6	0.0	56.5	-50.0	±0.4	3.835	367.9	0.66	321.2	0.30	351.5	0.91	255.5	0.07
Ni -7	-29.4	56.5	-40.5	±0.4	5.674	260.1	0.04	227.7	0.07	247.13	0.086	180.8	0.04
Ni -8	-47.6	56.5	-15.5	±0.4	8.142	184.6	0.02	161.9	0.06	175.3	0.11	127.3	0.14
Ni -9	-47.6	56.5	15.5	±0.4	11.716	132.7	0.02	117.1	0.03	125.9	0.33	90.3	0.14
Ni -10	-29.4	56.5	40.5	±0.4	16.83	92.7	0.09	81.9	0.02	89.0	0.17	64.3	0.05
Ni-11	-20.0	56.5	20.0	±0.4	23.827	65.4	0.10	57.7	0.02	62.7	0.13	45.7	0.12
Ni-12	20.0	56.5	20.0	±0.4	33.922	46.32	0.010	41.0	0.01	44.53	0.090	31.86	0.02
*Ni*13	20.0	56.5	-20.0	±0.4	48.609	32.45	0.012	28.7	0.03	30.84	0.016	22.38	0.02
Ni-14	-20.0	56.5	-20.0	±0.4	68.884	22.859	0.043 7	20.2	0.01	21.719	0.0054	15.83	0.03

TABLE S2: $MnCl_2$ Array

Contrast ID	X (mm)	Y (mm)	Z (mm)	positional accuracy (mm)	ICP measured $MnCl_2$ Concentration (mM)	NMR Measured T_1 @ 1.5 T (ms)	T_1 Standard Deviation (ms)	NMR Measured T_2 @ 1.5 T (ms)	T_2 Standard Deviation (ms)	NMR Measured T_1 @ 3.0 T (ms)	T_1 Standard Deviation (ms)	NMR Measured T_2 @ 3.0 T (ms)	T_2 Standard Deviation (ms)
Mn-1	0.0	16.5	50.0	±0.4	0.012	2 376	2.2	939.4	1.11	2 480	10.8	581.3	0.39
Mn -2	29.4	16.5	40.5	±0.4	0.019	2 183	1.2	594.3	0.32	2 173	14.7	403.5	0.55
Mn -3	47.6	16.5	15.5	±0.4	0.028	1 870	6.0	416.5	1.13	1 907	10.3	278.1	0.28
Mn -4	47.6	16.5	-15.5	±0.4	0.042	1 539	3.8	267.0	0.11	1 604	7.2	190.94	0.011
Mn -5	29.4	16.5	-40.5	±0.4	0.062	1 237	0.4	184.9	0.11	1 332	0.8	133.27	0.073
Mn -6	0.0	16.5	-50.0	±0.4	0.091	1 030	1.7	140.6	0.05	1 044	3.2	96.89	0.049
Mn -7	-29.4	16.5	-40.5	±0.4	0.131	752.2	1.12	91.76	0.029	801.7	1.70	64.07	0.034
Mn -8	-47.6	16.5	-15.5	±0.4	0.187	550.2	0.18	64.84	0.029	608.6	1.03	46.42	0.014
Mn -9	-47.6	16.5	15.5	±0.4	0.267	413.4	0.29	45.28	0.029	458.4	0.33	31.97	0.083
Mn -10	-29.4	16.5	40.5	±0.4	0.381	292.2	0.15	30.62	0.014	336.5	0.18	22.56	0.012
Mn -11	-20.0	16.5	20.0	±0.4	0.54	194.9	0.08	19.76	0.017	244.2	0.09	15.813	0.006 1
Mn -12	20.0	16.5	20.0	±0.4	0.762	160.2	0.23	15.99	0.012	176.6	0.09	11.237	0.005 7
Mn -13	20.0	16.5	-20.0	±0.4	1.08	106.4	0.02	10.47	0.006	126.9	0.03	7.911	0.003 7
Mn-14	-20.0	16.5	-20.0	±0.4	1.527	83.33	0.10	8.15	0.011	90.9	0.05	5.592	0.005 5

TABLE S3: Proton Density Array

Contrast ID	X (mm)	Y (mm)	Z (mm)	positional accuracy (mm)	D_2O %	NMR Measured T_1 @ 1.5 T (ms)	T_1 Standard Deviation (ms)	NMR Measured T_2 @ 1.5 T (ms)	T_2 Standard Deviation (ms)	NMR Measured T_1 @ 3.0 T (ms)	T_1 Standard Deviation (ms)	NMR Measured T_2 @ 3.0 T (ms)	T_2 Standard Deviation (ms)
PD-1	0.0	-23.5	50.0	±0.4	95	378.08	0.42	320.61	0.99	341.74	0.18	236.25	0.51
PD-2	29.4	-23.5	40.5	±0.4	90	371.76	0.10	311.21	0.52	336.69	0.08	233.59	0.27
PD-3	47.6	-23.5	15.5	±0.4	85	367.96	0.17	313.80	0.43	335.10	0.02	233.89	0.16
PD-4	47.6	-23.5	-15.5	±0.4	80	364.15	0.09	308.79	0.03	334.51	0.03	233.02	0.20
PD-5	29.4	-23.5	-40.5	±0.4	75	353.17	0.81	299.65	0.04	323.94	0.01	227.16	0.08
PD-6	0.0	-23.5	-50.0	±0.4	70	354.84	0.09	300.41	0.13	323.03	0.01	226.04	0.07
PD-7	-29.4	-23.5	-40.5	±0.4	65	365.43	0.18	310.05	0.28	343.58	0.05	240.48	0.12
PD-8	-47.6	-23.5	-15.5	±0.4	60	345.95	0.07	295.20	0.16	324.53	0.03	228.05	0.10
PD-9	-47.6	-23.5	15.5	±0.4	50	343.76	0.41	287.02	0.79	319.87	0.05	225.25	0.06
PD-10	-29.4	-23.5	40.5	±0.4	40	339.28	0.36	285.67	0.52	320.14	0.03	226.31	0.10
PD-11	-20.0	-23.5	20.0	±0.4	30	340.05	0.01	292.96	0.15	316.07	0.08	224.48	0.03
PD-12	20.0	-23.5	20.0	±0.4	20	328.78	0.08	284.15	0.06	309.31	0.02	220.68	0.09
PD-13	20.0	-23.5	-20.0	±0.4	10	329.23	0.05	287.47	0.05	311.68	0.03	224.09	0.06
PD-14	-20.0	-23.5	-20.0	±0.4	0	331.16	0.33	320.61	0.99	305.08	0.04	219.06	0.06

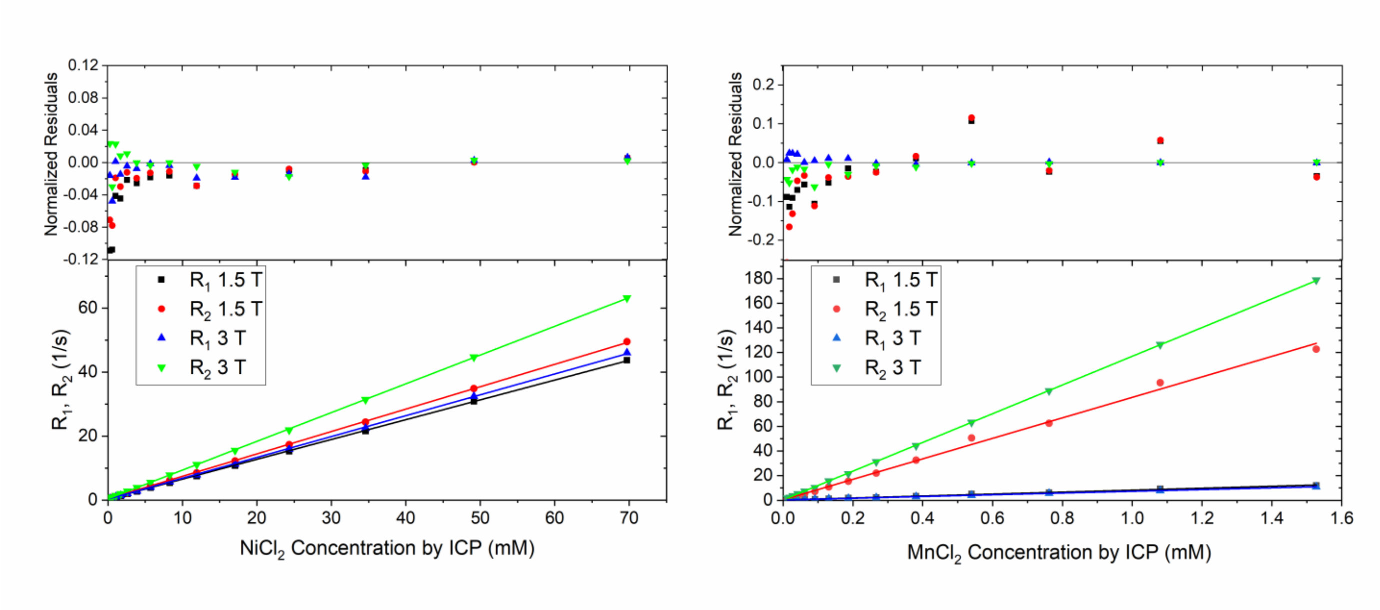

FIGURE S2 Relaxation rates as a function of paramagnetic salt concentration measured by inductively coupled Plasma (ICP) mass spectrometry at 1.5 T and 3.0 T. The solid lines are fits assuming a linear increase in relaxation rate with concentration and a zero-concentration intercept given by the measured values for high purity water.

TABLE S4: Water proton spin relaxivities determined from the slope of the data in Figure S2

**Relaxivities**	r_1 (20 °C, 1.5 T) mM^{-1}s^{-1}	r_2 (20 °C, 1.5 T) mM^{-1}s^{-1}	r_1 (20 °C, 3 T) mM^{-1}s^{-1}	r_2 (20 °C, 3 T) mM^{-1}s^{-1}
**$NiCl_2$**	0.619 ±0.002	0.700 ±0.002	0.652 ±0.002	0.899 ±0.002
**$MnCl_2$**	7.90 ±0.11	83.2 ±1.2	7.002 ±0.005	116.6 ±0.2
**Relaxation rates**	R_1 (20 °C, 1.5 T) s^{-1}	R_2 (20 °C, 1.5 T) s^{-1}	R_1 (20 °C, 3 T) s^{-1}	R_2 (20 °C, 3 T) s^{-1}
**ACS water**	0.367	0.436	0.316	0.398

3. Recommended Imaging Protocols

TABLE S5: Isotropic Volume Series

Isotropic Volume Series	GE	Philips	Siemens
**Sequence**	3D/SPGR	3D/SPGR	3D/RF spoiled GRE
**Scan Plane**	COR	COR	COR
**Scan Options**	EDR	3D FFE; Fast = none	
**Section Thickness / Gap (mm)**	1	1	1
**TR (ms)**	5.9	10.0	6.3
**TE (ms)**	1.4 (minimum)	4.0	1.9
**TI Values (ms)**			
**Flip Angle (deg)**	10	10	10
**ETL**	1	1	1
**Number of Averages**	1	1	1
**Matrix (FE)**	256	256	256
**Matrix (PE)**	256	256	256
**Matrix (SE) / # of Slices**	256	256	256
**Pixel Bandwidth (Hz)**	488	434	650
**Bandwidth (kHz) - GE**	62.5		
**FOV (FE, mm)**	250	250	250
**FOV (PE, mm)**	250	250	250
**Pixel Size (mm x mm)**	0.98 x 0.98	0.98 x 0.98 x 0.98	0.98 x 0.98
**PE Direction**	RL	RL	RL
**Notes**	Resolutions are important for each of these protocols. Keep resolution and adjust TE/TR as necessary.<br><br>Repeat for each plane (cor, sag, ax).	Resolutions are important for each of these protocols. Keep resolution and adjust TE/TR as necessary.<br><br>Repeat for each plane (cor, sag, ax).	Resolutions are important for each of these protocols. Keep resolution and adjust TE/TR as necessary.<br><br>Repeat for each plane (cor, sag, ax).
**Series**	Isotropic Volume	Isotropic Volume	Isotropic Volume
**Approx. Acq. Time per Setting (min)**	6.4	11.0	6.53
**# Settings**	1	1	1
**Total Time for this Series (min)**	6.4	11.0	6.53

TABLE S6: Section Thickness Series

Section Thickness Series	GE	Philips	Siemens
**Sequence**	2D/FSE	2D/SE	2D/TSE
**Scan Plane**	COR	COR	COR
**Scan Options**	EDR	MS SE; Fast=TSE	
**Section Thickness / Gap (mm)**	3/3, 5/5	3/3, 5/5	3/3, 5/5
**TR (ms)**	5000	2000	5000
**TE (ms)**	60	36	63
**TI Values (ms)**			
**Flip Angle (deg)**		90	
**ETL**	8	8	8
**Number of Averages**	2	1	2
**Matrix (FE)**	512	512	512
**Matrix (PE)**	256	256	256
**Matrix (SE) / # of Slices**	1	1	1
**Pixel Bandwidth (Hz)**	244	394	184
**Bandwidth (kHz) - GE**	62.5		
**FOV (FE, mm)**	250	250	250
**FOV (PE, mm)**	250	250	250
**Pixel Size (mm x mm)**	0.49 x 0.98	0.49 x 0.98	0.49 x 0.98
**PE Direction**	RL	RL	RL
**Notes**	Two series: one at 3mm slice and one at 5mm slice	Two series: one at 3mm slice and one at 5mm slice	Two series: one at 3mm slice and one at 5mm slice
**Series**	Section Thickness	Section Thickness	Section Thickness
**Approx. Acq. Time per Setting (min)**	5.3	1.5	2.47
**# Settings**	2	2	2
**Total Time for this Series (min)**	10.6	3.0	4.94

TABLE S7: Resolution Inset Series

Resolution Inset Series	GE	Philips	Siemens
**Sequence**	2D/FSE	2D/SE	2D/TSE
**Scan Plane**	COR	COR	COR
**Scan Options**	EDR	MS SE; Fast=TSE	
**Section Thickness / Gap (mm)**	4	4	4
**TR (ms)**	5000	5000	5000
**TE (ms)**	60	60	63
**TI Values (ms)**			
**Flip Angle (deg)**		90	
**ETL**	8	8	8
**Number of Averages**	2	2	2
**Matrix (FE)**	512	512	512
**Matrix (PE)**	512	504	512
**Matrix (SE) / # of Slices**	1	1	1
**Pixel Bandwidth (Hz)**	244	394	227
**Bandwidth (kHz) - GE**	62.5		
**FOV (FE, mm)**	250	250	250
**FOV (PE, mm)**	250	250	250
**Pixel Size (mm x mm)**	0.49 x 0.49	0.49 x 0.5	0.49 x 0.49
**PE Direction**	RL	RL	RL
**Notes**	Acquire 3 sections in one acquisition, spaced appropriately to cover the three resolution inserts (if extra coffins exist). Acquire both plate and coffin insets.	Acquire 3 sections in one acquisition, spaced appropriately to cover the three resolution inserts (if extra coffins exist). Acquire both plate and coffin insets.	Acquire 3 sections in one acquisition, spaced appropriately to cover the three resolution inserts (if extra coffins exist). Acquire both plate and coffin insets.
**Series**	Resolution Inset	Resolution Inset	Resolution Inset
**Approx. Acq. Time per Setting (min)**	10.7	10.6	5.27
**# Settings**	2	2	2
**Total Time for this Series (min)**	21.4	21.2	10.54

TABLE S8: Proton Density and Signal to Noise Series

PD & SNR Series	GE	Philips	Siemens
**Sequence**	2D/SE	2D/SE	2D/SE
**Scan Plane**	COR	COR	COR
**Scan Options**	EDR	MS SE; Fast=none	
**Section Thickness / Gap (mm)**	6	6	6
**TR (ms)**	5000	5000	5000
**TE (ms)**	10	10	10
**TI Values (ms)**			
**Flip Angle (deg)**		90	
**ETL**	1	1	1
**Number of Averages**	1	1	1
**Matrix (FE)**	256	256	256
**Matrix (PE)**	192	192	192
**Matrix (SE) / # of Slices**	1	1	1
**Pixel Bandwidth (Hz)**	244	213	153
**Bandwidth (kHz) - GE**	31.2		
**FOV (FE, mm)**	250	250	250
**FOV (PE, mm)**	200 (0.8 PFOV)	250	250
**Pixel Size (mm x mm)**	0.98 x 0.98	0.98 x 1.3	0.98 x 0.98
**PE Direction**	RL	RL	RL
**Notes**	Acquire twice and use NEMA MS 1-2008 Method 1 for SNR measurement.	Acquire as single-series, but two dynamics.	Acquire twice and use NEMA MS 1-2008 Method 1 for SNR measurement.
**Series**	PD & SNR	PD & SNR	PD & SNR
**Approx. Acq. Time per Setting (min)**	13.8	16.0	16.07
**# Settings**	2	2	2
**Total Time for this Series (min)**	27.7	32.0	32.14

TABLE S9: T_1 Inversion Recovery Series

T1 - VTI Series	GE	Philips	Siemens
**Sequence**	2D/FSE-IR	2D/IR-SK	2D/TSE-IR
**Scan Plane**	COR	COR	COR
**Scan Options**	EDR	2D IR; Fast=TSE	
**Section Thickness / Gap (mm)**	6	6	6
**TR (ms)**	4500	4500	4500
**TE (ms)**	Min Full (7.6)	7	6.9
**TI Values (ms)**	50, 75, 100, 125, 150, 250, 1000, 2000, 3000	35, 75, 100, 125, 150, 250, 1000, 1500, 2000, 3000	35, 75, 100, 125, 150, 250, 1000, 1500, 2000, 3000
**Flip Angle (deg)**			
**ETL**	3	6	6
**Number of Averages**	1	1	1
**Matrix (FE)**	256	256	256
**Matrix (PE)**	192	252	192
**Matrix (SE) / # of Slices**	1	1	1
**Pixel Bandwidth (Hz)**	391	436	279
**Bandwidth (kHz) - GE**	50		
**FOV (FE, mm)**	250	250	250
**FOV (PE, mm)**	200 (0.8 PFOV)	250	250
**Pixel Size (mm x mm)**	0.98 x 0.98	0.98 x 0.98	0.98 x 0.98
**PE Direction**	RL	RL	RL
**Notes**	Min TI allowed on GE: 50 ms<br><br>Autoprescan with TI = 50 ms	Reconstruct Magnitude, Real, Imaginary images. Do "FullPrep" for each of these ten series.	Use SOS (sum-of-squares) multi-channel coil reconstruction, not ACC (adaptive coil combine). Known "line" artifacts result when using the ACC method.
**Series**	T1 VTI	T1 VTI	T1 VTI
**Approx. Acq. Time per Setting (min)**	4.0	3.5	3.02
**# Settings**	9	10	10
**Total Time for this Series (min)**	36.0	35.0	30.20

TABLE S10: **T_1 Variable Flip Angle Series**

**T1 - VFA Series**	**GE**	**Philips**	**Siemens**
**Sequence**	3D/FSPGR	3D/SPGR	3D/RF spoiled GRE
**Scan Plane**	COR	COR	COR
**Scan Options**	EDR/Z2	3D FFE; Fast=none	
**Section Thickness / Gap (mm)**	3/0	6/0	6/0 (or 3/0 if error)
**TR (ms)**	Min (6.0)	6.6	6.6
**TE (ms)**	Min (1.4)	1.8	2.44
**TI Values (ms)**			
**Flip Angle (deg)**	2, 5, 10, 20, 25, 30	2, 5, 10, 20, 25, 30	2, 5, 10, 20, 25, 30
**ETL**	1	1	1
**Number of Averages**	4	4	4
**Matrix (FE)**	256	256	256
**Matrix (PE)**	192	192	192
**Matrix (SE) / # of Slices**	34	28	32
**Pixel Bandwidth (Hz)**	488	904	280
**Bandwidth (kHz) - GE**	62.5		
**FOV (FE, mm)**	250	250	250
**FOV (PE, mm)**	250	250	250
**Pixel Size (mm x mm)**	0.98 x 0.98	0.98 x 1.3 x 6	0.98 x 0.98
**PE Direction**	RL	RL	RL
**User CVs**	Turbo = 0		
**Notes**	1) Yields 30 3 mm sections.<br>2) Autoprescan with FA=15.<br>3) Ensure gain settings do not vary between series, i.e., use Manual Prescan.<br>4) Include fiducial spheres above & below T1 spheres in scan volume.<br>5) Using Turbo=0 should maintain the same TE/TR for each FA.	1) Reconstructed at 3 mm.<br>2) Ensure gain settings do not vary between series, i.e., use MPS. **Autoprescan using 15 deg FA.**<br>3) Include fiducial spheres above & below T1 spheres in scan volume.	1) Reconstructed at 3 mm.<br>2) Ensure gain settings do not vary between series, i.e., use MPS. **Autoprescan using 15 deg FA.**<br>3) Include fiducial spheres above & below T1 spheres in scan volume.
**Series**	T1 VFA	T1 VFA	T1 VFA
**Approx. Acq. Time per Setting (min)**	2.6	3.0	1.23
**# Settings**	7	7	7
**Total Time for this Series (min)**	18.2	21.0	8.61

TABLE S11: ***T_2* Series**

T2 Series	GE	Philips	Siemens
**Sequence**	2D/SE	2D/SE	2D/se_mc
**Scan Plane**	COR	COR	COR
**Scan Options**	EDR	2D SE; Fast=none	
**Section Thickness / Gap (mm)**	6	6	6
**TR (ms)**	5000	5000	5000
**TE (ms)**	15, 30, 45, 60;<br>25, 50, 75, 100;<br>40, 80, 120, 160	11 ms x 16 echoes	10 to 320 ms by 10 ms
**TI Values (ms)**			
**Flip Angle (deg)**			
**ETL**	1	1	1
**Number of Averages**	1	1	1
**Matrix (FE)**	256	256	256
**Matrix (PE)**	192	192	192
**Matrix (SE) / # of Slices**	1	1	1
**Pixel Bandwidth (Hz)**	156	172	227
**Bandwidth (kHz) - GE**	20.0		
**FOV (FE, mm)**	250	250	250
**FOV (PE, mm)**	200 (0.8 PFOV)	250	250
**Pixel Size (mm x mm)**	0.98 x 0.98	0.98 x 1.3	0.98 x 0.98
**PE Direction**	RL	RL	RL
**Notes**	Autoprescan with TE = 15 ms.<br><br>Ensure gain settings do not vary between series, i.e., use Manual Prescan.	Ensure gain settings do not vary between series, i.e., use MPS.	
**Series**	T2	T1 VFA	T1 VFA
**Approx Acq Time per Setting (min)**	13.8	16.0	16.1
**# Settings**	3	1	1
**Total Time for this Series (min)**	41.5	16.0	16.1

4. Materials Database

TABLE S12: **Starting Chemicals for Contrast Fluids**

Chemical	Purity	Molecular Weight (g/mol)
$CuSO_4•5H_2O$	Technical grade	249.68
$NiCl_2•6H_2O$	99.999% trace metal basis	237.69
$MnCl_2•4H_2O$	99.99% trace metal basis	197.91
D_2O	D, 99.8%;<br>chemical purity: 99.5%	20.03
H_2O	Deionized	18.02
Erioglaucine disodium salt	unknown	792.85
Tartrazine	≥85% Dye content	534.36
Allura Red AC	80% Dye content	496.42

Density of H_2O at 20 °C: 0.9982 g/cm^3

Density of D_2O at 20 °C: 1.105 g/cm^3

Fiducial solution [$CuSO_4$]:

Fiducial solutions were made at 20 °C by dissolving crystalline $CuSO_4•5H_2O$ (Millipore-Sigma, St. Louis, MO, USA; Part number: 209198) into deionized water with color doped by erioglaucine disodium salt (FD&C Blue No. 1; Millipore-Sigma, St. Louis, MO, USA; Part number: 861146). The target concentration for Cu was 3.21 mM with a doping of 1.47 µM erioglaucine disodium salt. For making one liter of fiducial solution:

Cu: $\frac{0.00321\ mol}{L} * 1\ L * \frac{249.68\ g}{mol} = 0.8015\ g$

Erioglaucine disodium salt: $\frac{0.00000147\ mol}{L} * 1\ L * \frac{792.85\ g}{mol} = 0.0012\ g$

$NiCl_2$ Array:

$NiCl_2$ contrast array solutions were made at 20 °C from a stock solution of $NiCl_2•6H_2O$ (Millipore-Sigma, St. Louis, MO, USA; Part number: 203866) with a concentration of 101 mM Ni^{++}, also made at 20 °C. Each contrast solution was color doped with erioglaucine disodium salt and tartrazine (FD&C

Yellow No. 5; Millipore-Sigma, St. Louis, MO, USA; Part number: T0388). For making one liter of stock $NiCl_2$ solution:

Ni: $\frac{0.101\ mol}{L} * 1\ L * \frac{237.69\ g}{mol} = 24.0067\ g$

To color dope each dilution of stock $NiCl_2$ solution, the following amount of dye was needed per dilution (assuming 100 mL volume):

Erioglaucine disodium salt: $\frac{0.000035\ mol}{L} * 0.1\ L * \frac{792.85\ g}{mol} = 0.00277\ g$

Tartrazine: $\frac{0.0000524\ mol}{L} * 0.1\ L * \frac{534.36\ g}{mol} = 0.0028\ g$

Based on the stock solution concentration, the following volumes are necessary for each of the dilutions (based on $C_{initial}V_{initial} = C_{final}V_{final}$), assuming 100 mL final volume:

TABLE S13: **$NiCl_2$ Solutions**

$NiCl_2$ Contrast Number	Desired Concentration (mM)	Volume of stock solution needed (mL)
Ni-1	0.299	0.27
Ni-2	0.62	0.56
Ni-3	1.074	0.98
Ni-4	1.716	1.56
Ni-5	2.624	2.39
Ni-6	3.908	3.56
Ni-7	5.724	5.21
Ni-8	8.293	7.55
Ni-9	11.925	10.85
Ni-10	17.062	15.53
Ni-11	24.327	22.14
Ni-12	34.6	31.48
Ni-13	49.129	44.70
Ni-14	69.677	64.40

$MnCl_2$ Contrast Array:

$MnCl_2$ contrast array solutions were made at 20 °C from a stock solution of $MnCl_2$•$4H_2O$ (Millipore-Sigma, St. Louis, MO, USA; Part number: 221279) with a concentration of 25 mM, also

made at 20 °C. Each contrast solution was color doped with Allura Red AC (FD&C Red 40; Millipore-Sigma, St. Louis, MO, USA; Part number: 458848). For making one liter of stock $MnCl_2$ solution:

Mn: $\frac{0.025\ mol}{L} * 1\ L * \frac{197.91\ g}{mol} = 4.9478\ g$

To color dope each dilution of stock $MnCl_2$ solution, the following amount of dye was needed per dilution (assuming 100 mL volume):

Allura Red AC: $\frac{0.0001\ mol}{L} * 0.1\ L * \frac{496.42\ g}{mol} = 0.00496\ g$

Based on the stock solution concentration, the following volumes are necessary for each of the dilutions (based on $C_{initial}V_{initial} = C_{final}V_{final}$), assuming 100 mL final volume:

TABLE S14: **$MnCl_2$ Solutions**

$MnCl_2$ Contrast Number	Desired Concentration (mM)	Volume of stock solution needed (mL)
Mn-1	0.01206	0.0482
Mn-2	0.01989	0.0796
Mn-3	0.03076	0.1230
Mn-4	0.04663	0.1865
Mn-5	0.06879	0.2752
Mn-6	0.1001	0.4004
Mn-7	0.14442	0.5777
Mn-8	0.20705	0.8282
Mn-9	0.29565	1.1826
Mn-10	0.42094	1.6838
Mn-11	0.59814	2.3926
Mn-12	0.84873	3.3949
Mn-13	1.20313	4.8125
Mn-14	1.70431	6.8172

Proton Density Contrast Array [D_2O/H_2O]:

Proton density (PD) contrast array solutions were made at 20 °C from solutions of D_2O, H_2O and stock solution of $NiCl_2$ (noted previously in T_1 contrast array section). Each proton density contrast solution was made independently, and color doped with tartrazine.

To color dope each proton density contrast solution, the following amount of dye was needed per dilution (assuming 100 mL volume):

Tartrazine: $\frac{0.0001\ mol}{L} * 0.1\ L * \frac{534.36\ g}{mol} = 0.0053\ g$

To reduce T_1 of water for acceptable scan times, each proton density contrast solution is doped with 0.1 mL of $NiCl_2$ stock solution (101 mM) for a final concentration of (assuming 100 mL volume):

Ni: $0.1\ mL * 101\ mM = 100\ mL * x\ mM\ \rightarrow x = 0.101\ mM$

Because of the addition of water as part of the $NiCl_2$ stock solution, it is important to subtract that volume from the water used for the remainder of the proton density contrast solution.

Based on the stock solution concentration, the following volumes are necessary for each of the dilutions (based on $C_{initial}V_{initial} = C_{final}V_{final}$), assuming 100 mL final volume:

TABLE S15: **Proton Density Solutions**

PD Contrast Number	Desired H_2O/D_2O (%/%)	Volume of water after $NiCl_2$ stock solution (mL)
PD-1	5/95	4.9
PD-2	10/90	9.9
PD-3	15/85	14.9
PD-4	20/80	19.9
PD-5	25/75	24.9
PD-6	30/70	29.9
PD-7	35/65	34.9
PD-8	40/60	39.9
PD-9	50/50	49.9
PD-10	60/40	59.9
PD-11	70/30	69.9
PD-12	80/20	79.9
PD-13	90/10	89.9
PD-14	100/0	99.9

5. Fiducial Array/ Geometric Distortion Measurements

Figure S3 schematically shows the fiducial array analysis process. The input 3D gradient echo image is cropped after a manual coarse alignment of the ROIs to the fiducial spheres. The 3D cropped image is then convolved with a synthetic fiducial sphere mask. A fiducial sphere image is shown as a comparison to the mask. Both real and synthetic images have partial volume effects and finite k-space sampling structure (Gibbs ringing). A slice of the 3D convolution is shown along with lines scans that are fit with a Gaussian model to determine sphere centers.

Figure S4 shows the 10 mm ROIs after sphere location and after the translation, rotation, and scaling, required to minimize $\sum_i(\vec{R}_{ai} - \vec{R}_{pi})^2$, is applied. Here, $\vec{R}_{ai}$ is the apparent position and $\vec{R}_{pi}$, is the prescribed center position of the ith sphere. Once the sphere centers have been located, the integrated signal $I_i = \sum_j I_{ij}$, shown in Figure S4E, can be calculated as a measure of image uniformity. The sum of voxel intensities I_{ij} for the ith sphere is over all voxels, j, whose centers are within 6 mm from the sphere center. For the scan shown in Figure S4, the anterior and posterior spheres have distinctly lower signal. The sphere centers can be calculated with a variety of algorithms. Figure S4F shows a comparison between sphere centers determined by fitting the convolution profiles and by calculating the center of mass $\vec{R}_{ai} = \sum_j \vec{R}_{aj} I_{ij} / I_i$, where the sum is over the same the voxel set used to calculate the integrated intensity. As seen in Figure S4F, the agreement is within 0.04 mm.

Figure S5 shows the distortion analysis from a scan that has non-uniform gradient corrections. In this case, they appear to be only done in the scan plane. The corrected *x* and *z* distortions (in-plane distortion) have standard deviations of 0.11 mm and 0.22 mm, respectively, whereas the *y* distortion perpendicular to the scan plane has a standard deviation of 0.63 mm. A completely independent analysis on the same data set, which located the sphere centers by finding their boundary region, gave standard deviations in the in-plane *x*, *z* directions of 0.17 mm, 0.17 mm, respectively, and the out-off-plane *y* direction of 0.66 mm.

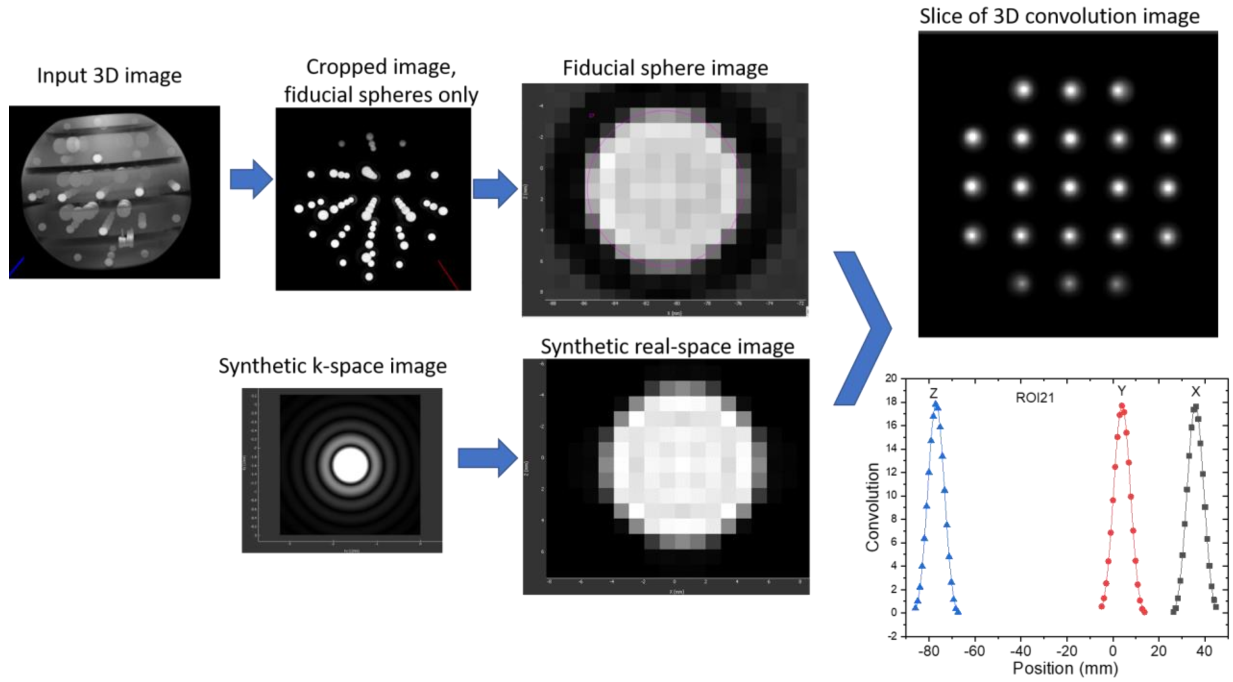

FIGURE S3 Schematic of the fiducial array analysis showing input 3D image of the system phantom, cropped image with just the fiducial spheres, image of fiducial sphere, synthetic k-space image and real-space image used as a convolution mask, slice of 3D convolution image, and convolution profiles with fits used to obtain sphere center.

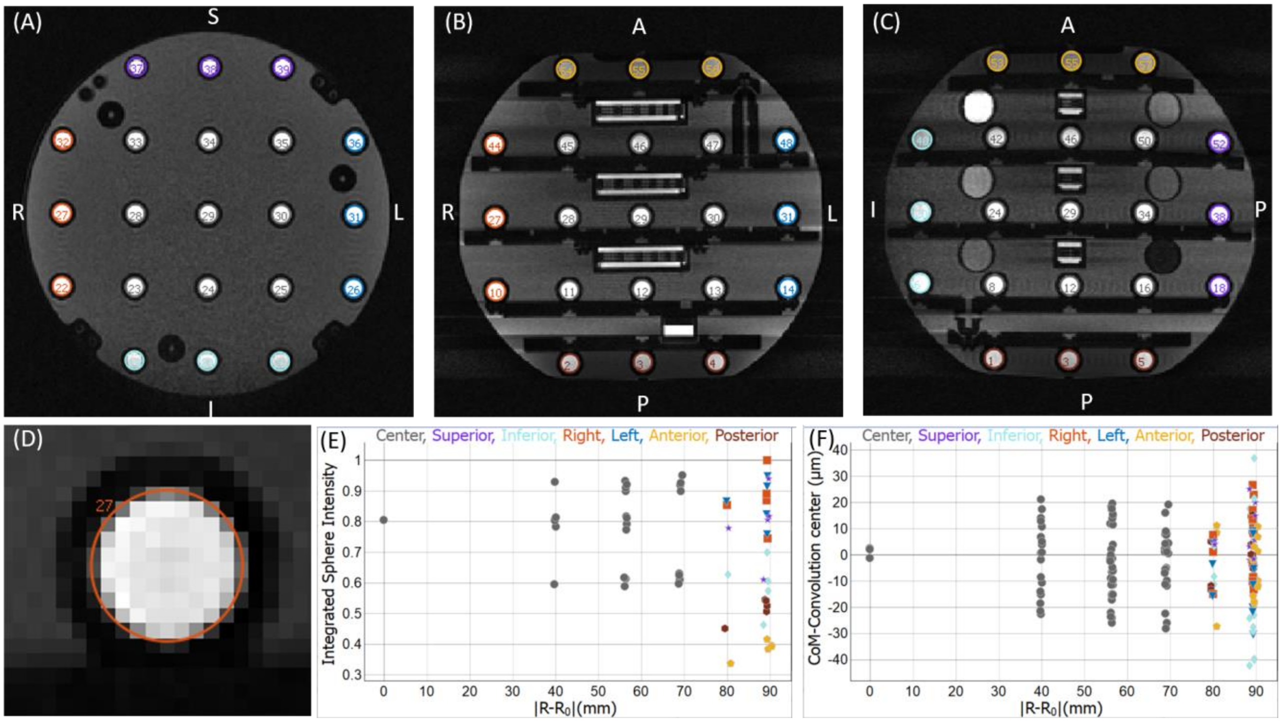

FIGURE S4 Fiducial analysis on a 1.5 T scanner with a gradient echo sequence. A, Coronal slice, B, axial slice, C, sagittal slice. D, magnified image of fiducial sphere image with ROI location after automated location. E, Normalized integrated intensity for all 57 fiducial spheres. F, Difference between center of mass and convolutional sphere centers.

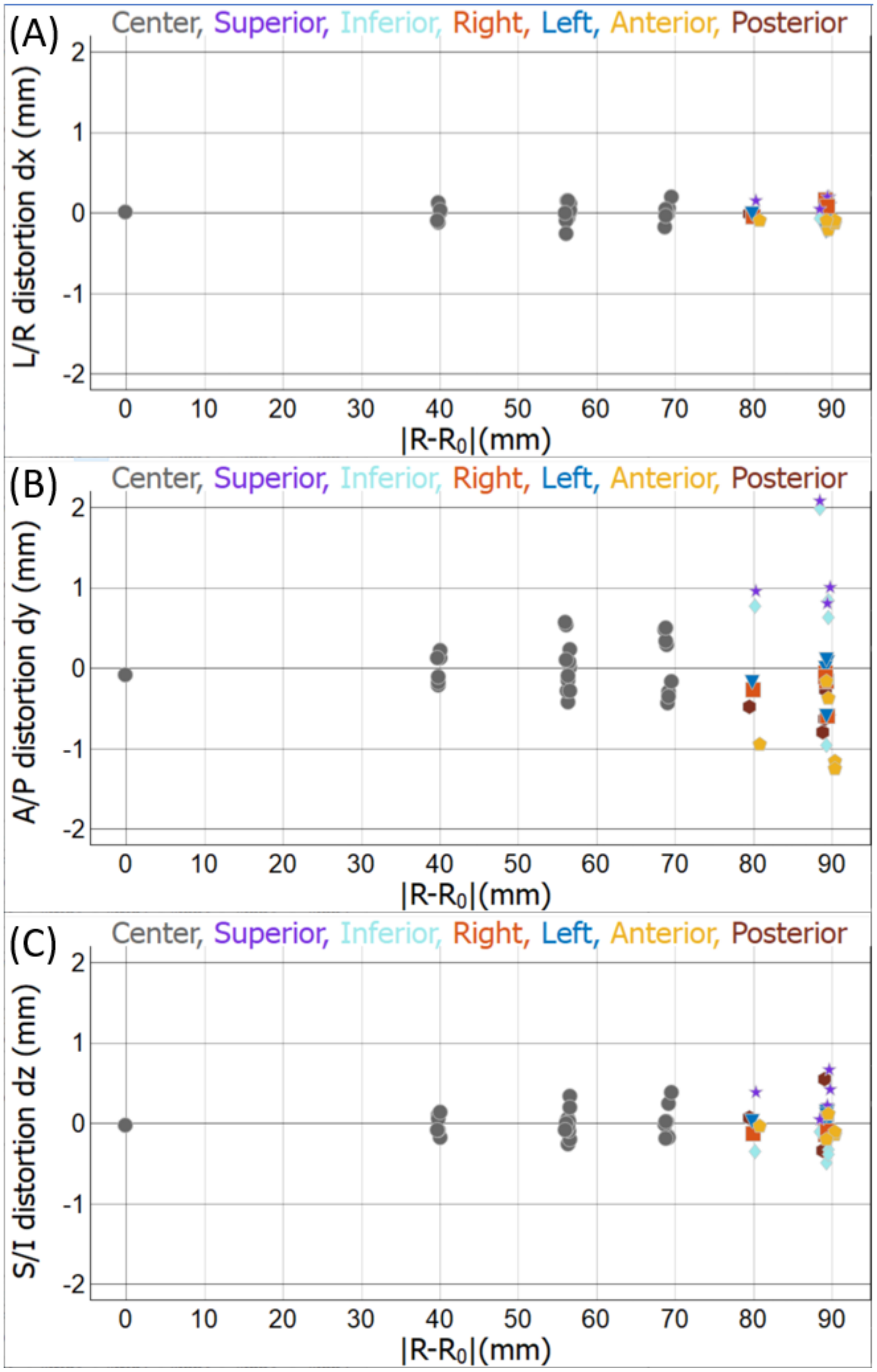

FIGURE S5 A, B, C, Geometric distortion of the 57 fiducial sphere centers in *x*, *y* and *z* directions.

6. NMR Calibration Measurements

Here we give an overview of SI-traceable NMR measurement protocols of proton relaxation times, fully described in NIST SP-250-97 (1) and available at https://www.nist.gov/programs-projects/magnetic-resonance-imaging-mri-biomarker-measurement-service. Briefly, NMR samples were prepared by injecting the contrast fluids into Teflon or flame sealed borosilicate glass capillaries for single measurements and long-term monitoring, respectively. Complete libraries containing 3 flame sealed capillaries of all solutions were made. Temperature measurements were made using a fiberoptic thermometer placed 15 mm from the center of the RF coils. The fiberoptic thermometer was calibrated against two NIST-calibrated platinum resistance thermometers. The uncertainty in the temperature is typically ± 0.3 °C.

T_1 Measurement

The longitudinal relaxation time, T_1, is defined as the exponential recovery time of the local magnetization along the applied field direction after it is driven out of equilibrium. NMR inversion recovery (IR) experiments were conducted by monitoring longitudinal relaxation of ^{1}H nuclei at a frequency corresponding to 128 MHz (± 0.1 MHz) and 64 MHz (± 0.1 MHz) at 20 °C. The NMR IR experiment utilized a composite 180° RF pulse for inversion to minimize errors relating to the calculation of the 90° and 180° times. Each reported T_1 value is obtained from a collection of 20 inversion time (*TI*) spectra, where *TI* is the time between the end of the 180° RF inversion pulse and the start of 90° RF pulse, which tips the spins back into the transverse plane for data acquisition. The *TI* values are chosen to approximately span the range from 0.01 T_1 to 5 T_1.

Each inversion time spectrum is the Fourier transform of the average of eight phase-cycled free-induction decay measurements. The delay between measurements is required to be greater than five times the T_1 value, if it is determined that the delay between measurements is shorter than five times the T_1 value, the experiment is repeated with an appropriate length delay. The complex spectra were phase shifted so that the real component gave a symmetric Lorentzian peak. Peak integration was determined by fitting the spectrum with a Lorentzian and numerically integrating ±10 linewidths. Integrated values from each inversion time spectra, shown in Figure S6, were fit using a nonlinear least-squares procedure to an exponential function of the form:

$$S(TI) = \mathrm{A}\left(1 - (1 + \delta)e^{-\frac{TI}{T_1}}\right).$$

where *S(TI)* is the integrated signal intensity for an inversion time *TI* and δ is a fit parameter that indicates the degree of inversion in the peak integration data with an ideal value of $\delta = 1$. Each experiment was repeated three times without adjusting the sample, nor repeating the shim. Fitted T_1 times were then averaged together, and deviation is reported for the three measurements from the mean T_1 value (SD_{3R}). NMR details of the experiments can be found in (2) with composite pulse details found in (3).

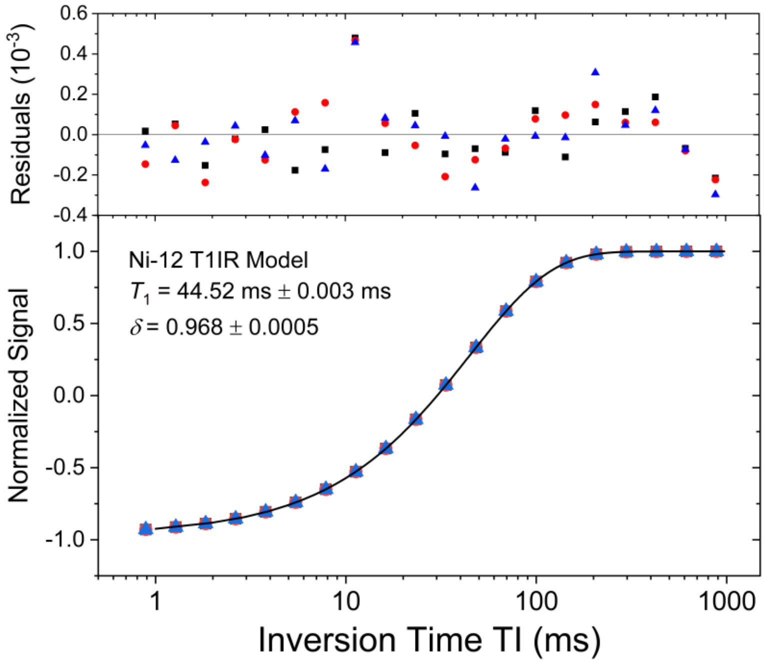

FIGURE S6 NMR signal for Ni-12 versus inversion time for the T1-IR protocol along with fits to the model described in the text. Data for three consecutive measurements at 3 T and 20 °C, are shown along with the residuals for each measurement (top plot). The errors listed for T_1 and the inversion efficiency are the standard deviation of the 3 values obtained for each of the measurements.

T_2 Measurement

NMR Carr-Purcell-Meiboom-Gill (CPMG) experiments were conducted by monitoring transverse relaxation of ^{1}H nuclei at a frequency corresponding to 128 MHz (± 0.1 MHz) and 64 MHz (± 0.1 MHz) at 20 °C. The NMR experiment consists of a 90° RF pulse to tip the spins, about the x-axis, into the transverse plane and then a train of 180° RF refocusing pulses about the y-axis until the desired acquisition time, $t_a = n\tau_{rt}$, is reached. Here, n is the number of 180° RF refocusing pulses, $\tau_{rt} = 2\tau_{cp} + t_{180}$ is the refocusing time, τ_{cp} = 1 ms is the delay before and after the 180° pulse, and t_{180} is the duration of the 180° pulse. Each reported T_2 value is obtained from a collection of 20 CPMG

sequences where the number of refocusing pulses is increased to vary t_a. The t_a values are logarithmically spaced with a maximum time that insures observation of at least three decades of signal decrease. For these measurements, the signal-to-noise ratio varied from 2000 to 5000.

Each CPMG spectrum is the Fourier transform of the average of eight phase-cycled free-induction decay measurements. The delay between measurements is required to be greater than five times the T_1 value (as determined by NMR-IR measurements), if it is determined that the delay between measurements is shorter than five times the T_1 value, the experiment is repeated with an appropriate length delay. The complex spectra were phase shifted so that the real component gave a symmetric Lorentzian peak. Peak integration was determined by fitting the spectrum with a Lorentzian and numerically integrating ±10 linewidths. Integral values from each CPMG spectra $S(t_a)$ vs. acquisition time are shown in Figure S7 along with nonlinear least-squares fits to an exponential function of the form:

$$S(t_a) = S_0 e^{-t_a/T_2} .$$

Each experiment was repeated three times without adjusting the sample, nor repeating the shim. Fitted T_2 times were then averaged together, and deviation is reported for the three measurements from the mean T_2 value (SD_{3R}). The transverse relaxation time, T_2, will be dependent on the refocusing time, $2\tau_{cp} + t_{180}$, and, therefore, T_2 must be reported with the refocusing time used. NMR details of NMR-CPMG experiments can be found in (2) with original references (4-6).

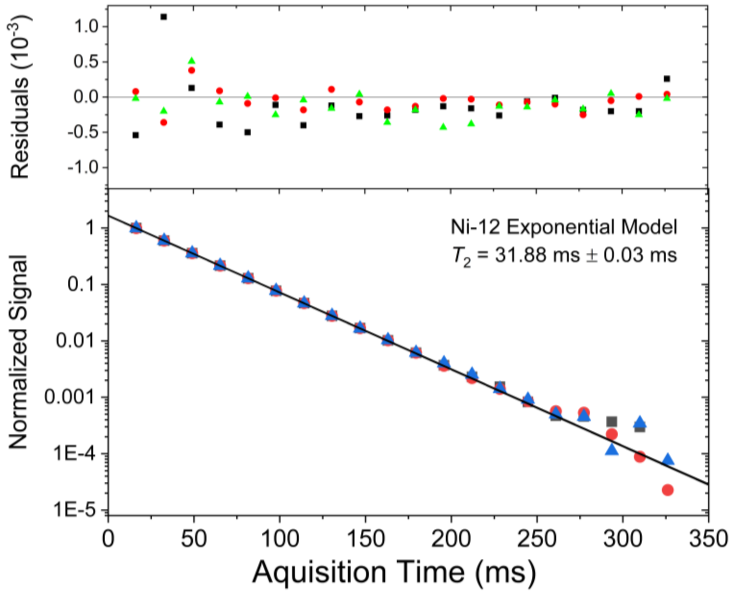

FIGURE S7 NMR signal from Ni-12 using a CPMG sequence as a function of acquisition time along with exponential fits. Data for three consecutive measurements at 3 T and 20 °C, are shown along with the residuals for each measurement (top plot). The errors listed for T_2 are the standard deviation of the 3 values obtained for each of the measurements.

7. Field, Temperature, and Time Stability

The proton relaxation times, as measured by NMR, of the MR-parameter solutions exhibit considerable magnetic field and temperature dependence. The field dependencies of T_1, T_2, taken from the measured relaxation times given in Tables S1, S2, are shown in Figure S8. Here, the normalized

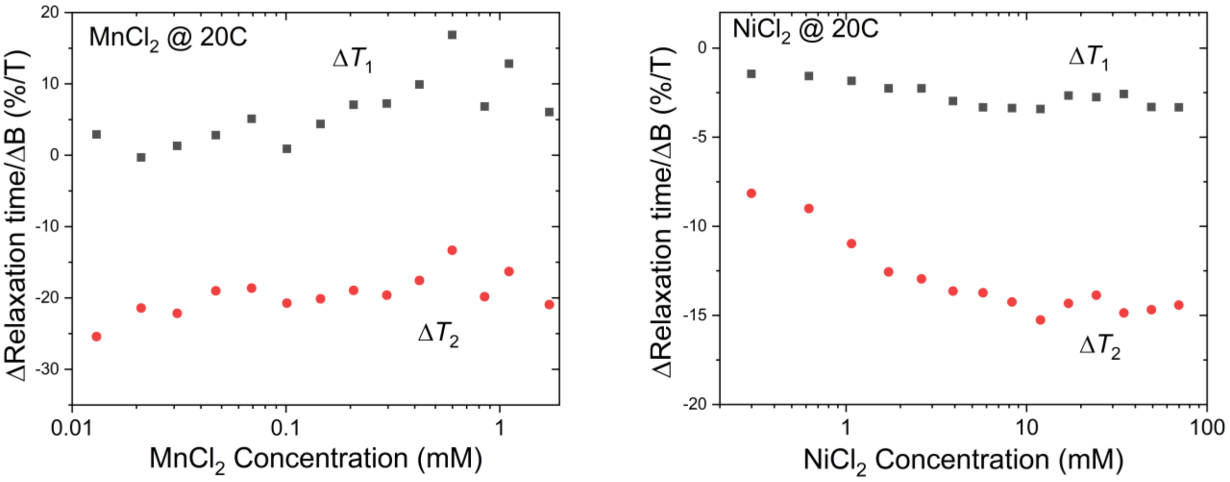

FIGURE S8 Magnetic field dependence of T_1 and T_2 for the $MnCl_2$ and $NiCl_2$ arrays.

change in proton relaxation time per unit field, in units of %/T, is given by $\frac{100}{1.5T}\frac{T_{1,2}(3T)-T_{1,2}(1.5T)}{T_{1,2}(1.5T)}$ and is only a course measurement of the field dependence. Note that the $NiCl_2$ T_1 times decrease with increasing field, while the $MnCl_2$ T_1, along with most tissues, increase with field. Given the large field dependence, especially of T_2, small variations in imaging field of commercial scanners operating at nominally the same field values, must be taken into account.

An example of the temperature dependence at 3.0 T and 4-year stability of the Ni-12 solution is shown in Figure S9. The variations in T_1, T_2 over the typical MRI bore temperature range (18 °C to 26 °C) are 1.3 % and 1.6 %, respectively. The repeatability in the measurement of the same sample for 3 measurements made 49 months apart is less than 0.1 %.

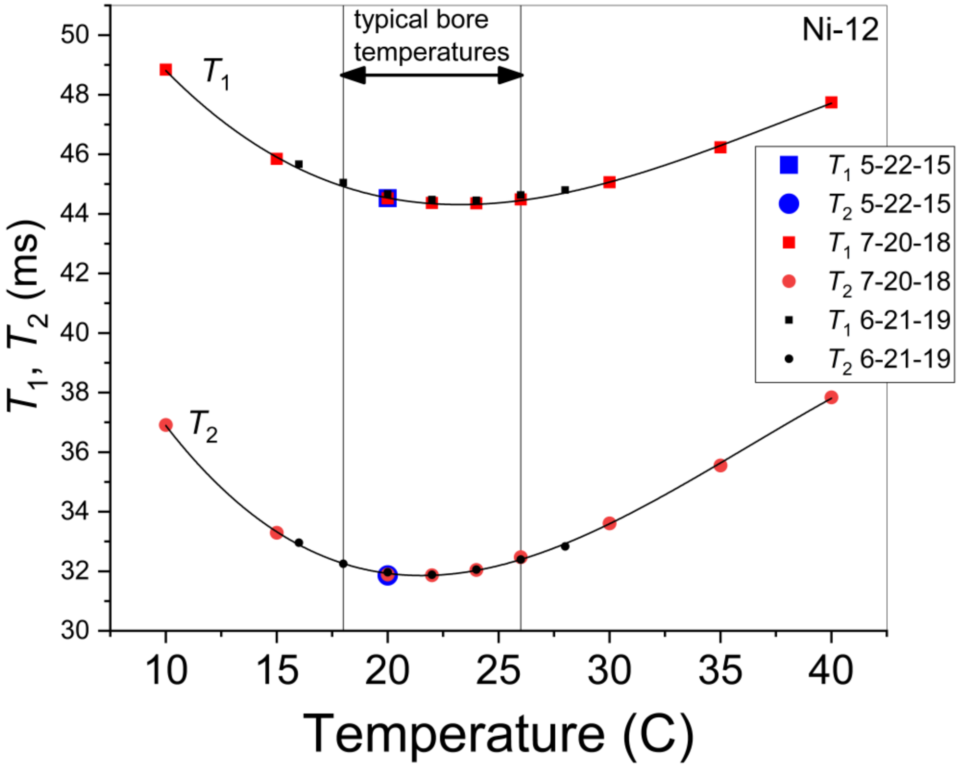

FIGURE S9 Temperature dependence of T_1, T_2 for the Ni-12 solution measured in a metrology NMR at 3.0 T. The plot shows data from a flame-sealed borosilicate-capillary library sample over the course of 4 years.

The temperature variations of the proton spin relaxation rates in the $NiCl_2$ array are complex, as seen in Figure S10, exhibiting a minimum around 20 °C for the higher concentrations, while exhibiting a more linear behavior for lower concentrations. The minimum in T_1 is characteristic of the resonance frequency, in this case 128 MHz, being aligned with a characteristic fluctuation rate. The minimum in relaxation times is useful to provide less temperature variation, but it is more difficult to provide thermal correction formulas since the form of the temperature variation changes with concentration and field.

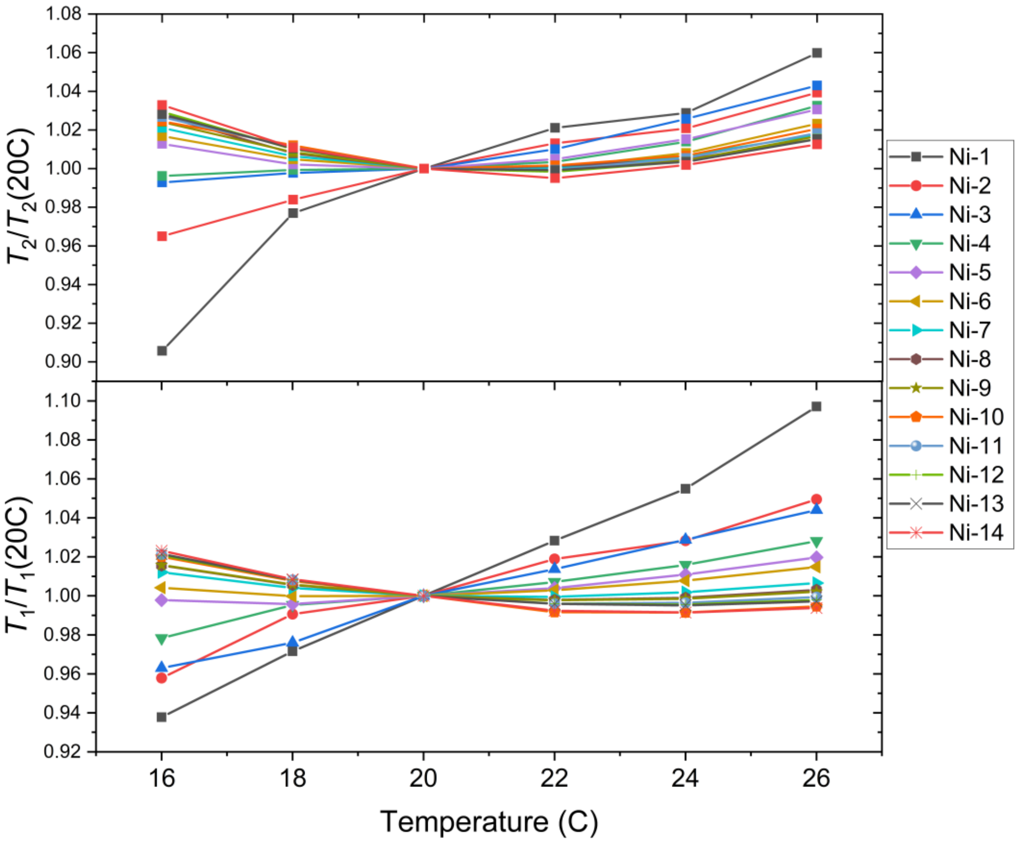

FIGURE S10 Normalized relaxation times versus temperature for the $NiCl_2$ array at 3.0 T.

The $MnCl_2$ solutions exhibit a linear variation of T_1 and T_2 over the measurement range of 16 °C to 26 °C, with both T_1 and T_2 increasing with temperature. T_1 and T_2 have temperature coefficients of ~ 3%/C and 2%/°C, respectively, at 3.0 T as shown in Figure S11. The $CuSO_4$ fiducial solution exhibits a similar linear dependence of T_1 and T_2 on temperature, as seen in Figure S12, with temperature coefficients of 2.72 %/°C and 2.65 %/°C, respectively

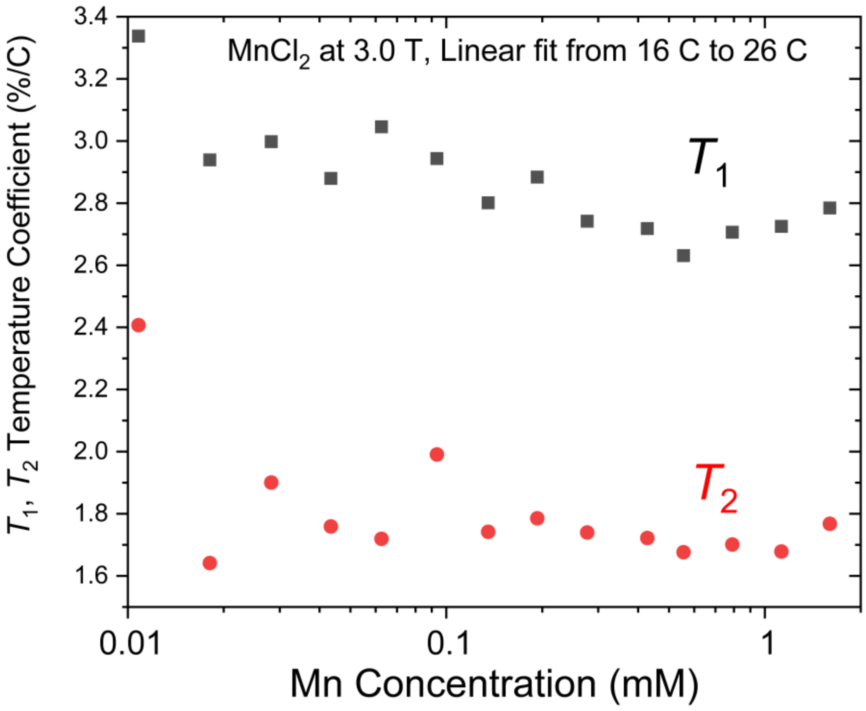

FIGURE S11 Temperature coefficient of spin relaxation times for the $MnCl_2$ array at 3.0 T.

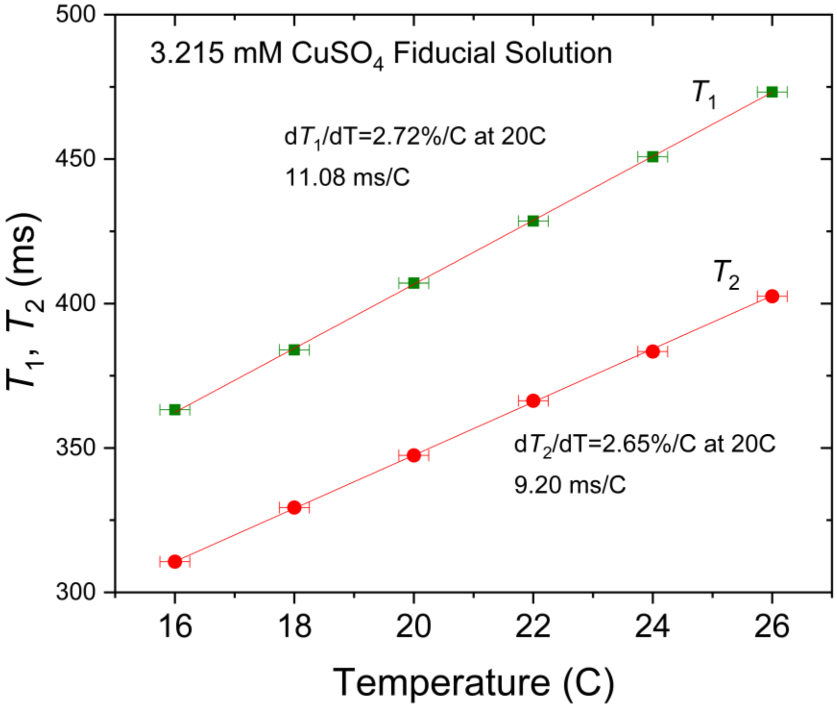

FIGURE S12 Relaxation times versus temperature for the $CuSO_4$ fiducial solution at 3.0 T.

Figure S13 shows several T_1-IR measurements of the $NiCl_2$ array, plotted as a deviation from the NMR reference values at 20 °C, in the system phantom protypes in various 3 T scanners over the course of 7 years. The error bars indicate the standard errors in the nonlinear least square fits, while the grey bar indicate the uncertainty of the values given typical bore temperature can range from 10 °C to 22 °C. The average standard deviation of the middle 10 cells is 1.5%, which give an upper limit to the phantom stability over 7 years. A more rigorous stability protocol has been implemented for commercial system phantoms that are available from the NIST/NIBIB Phantom Lending Library.

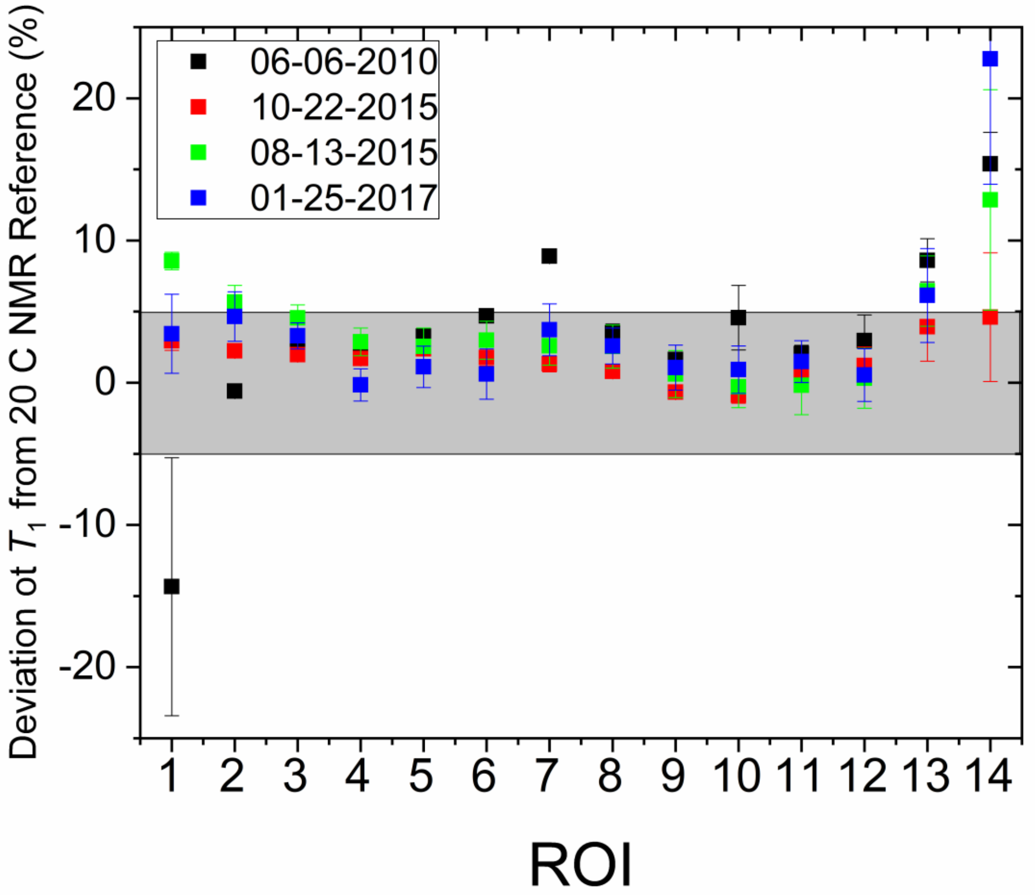

FIGURE S13 Deviation of T_1-IR values from NMR reference values at 3 T over the course of 7 years. The gray bar indicates the expected range of values given a phantom temperature that can vary between 18 °C and 22 °C.

8. Material Stability

Properties of materials used in the phantom were studied to determine water uptake and dimensional stability. A plot of water uptake is shown in Figure S14 and dimensional changes during water absorption are shown in Figure S15 for a variety of plastics. Water uptake can lead to undesirable water loss/exchange with the surrounding environment and to geometric distortion. Based on this data, PVC and PP were selected for the fiducial and contrast arrays, respectively, and PPS was selected for the components requiring high dimensional tolerance (the structural plates). Note, since there are no standard properties for many plastics, this data is for guidance only. Each specific material used in the phantom must be tested.

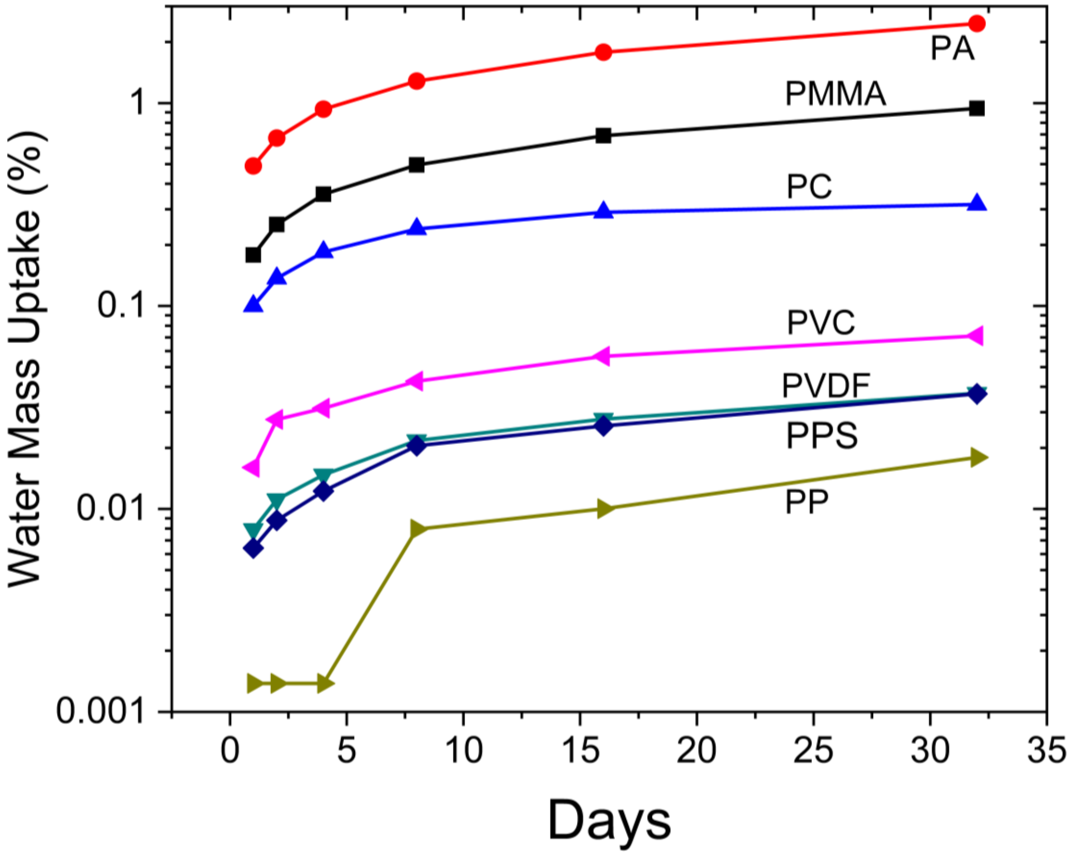

FIGURE S14 **Water mass uptake for various plastics: nylon/polyamide (PA), poly(methyl methacrylate) (PMMA), polycarbonate (PC), polyvinyl chloride (PVC), polyvinylidene fluoride (PVDF), polyphenylene sulfide (PPS), polypropylene (PP). The samples were 25 mm diameter, 6 mm thick disks.**

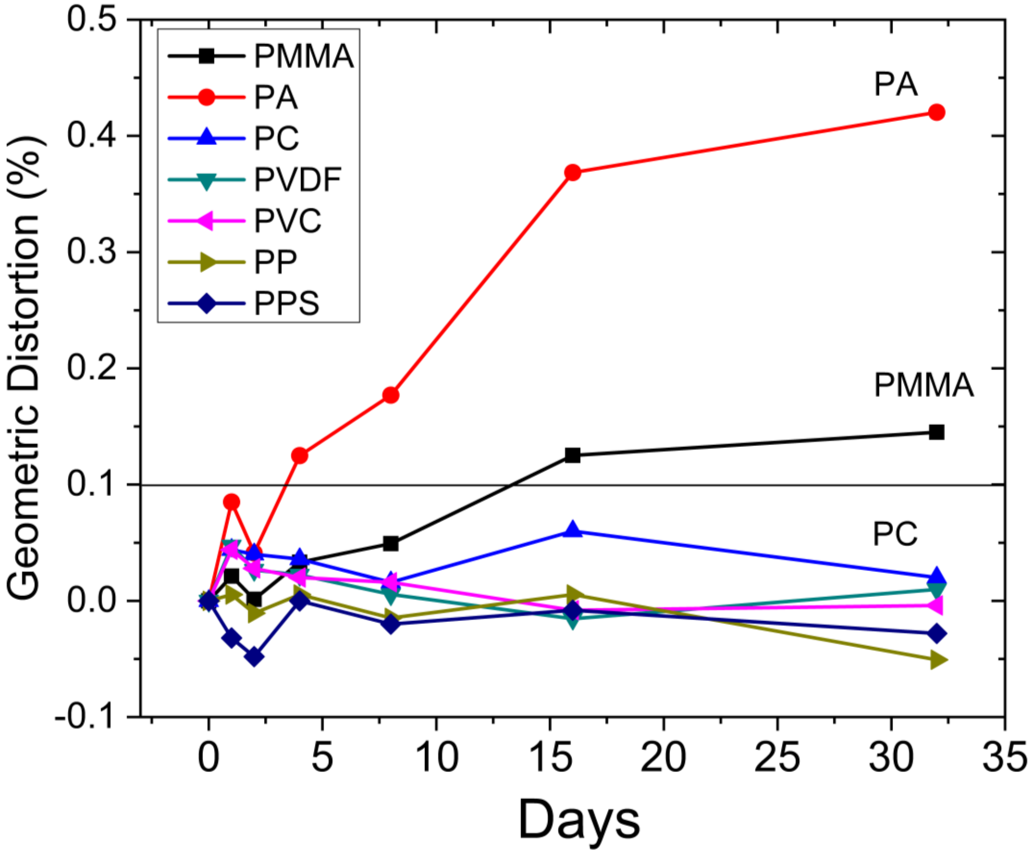

FIGURE S15 Geometric distortion during water soaking of the same samples used in Figure S14. The horizontal line indicates the threshold for maintaining the specified geometric distortion of the phantom plates.

Spin relaxation rates may be sensitive to the solution pH, which may be a source of instability if the pH is not controlled. Figure S16 shows a study of relaxation times at 3T, 20 °C, for several $NiCl_2$ solutions as a function of pH. The solutions as prepared have a pH near 5.0. The pH was then modified by addition of small amounts of HCl and NaOH. The relaxation rates were stable over a wide range of pH indicating that small changes in the pH due to sample transfers and handling will not have a substantial effect on the spin relaxation times. The solutions became unstable and the salts precipitated out at a pH of 10.

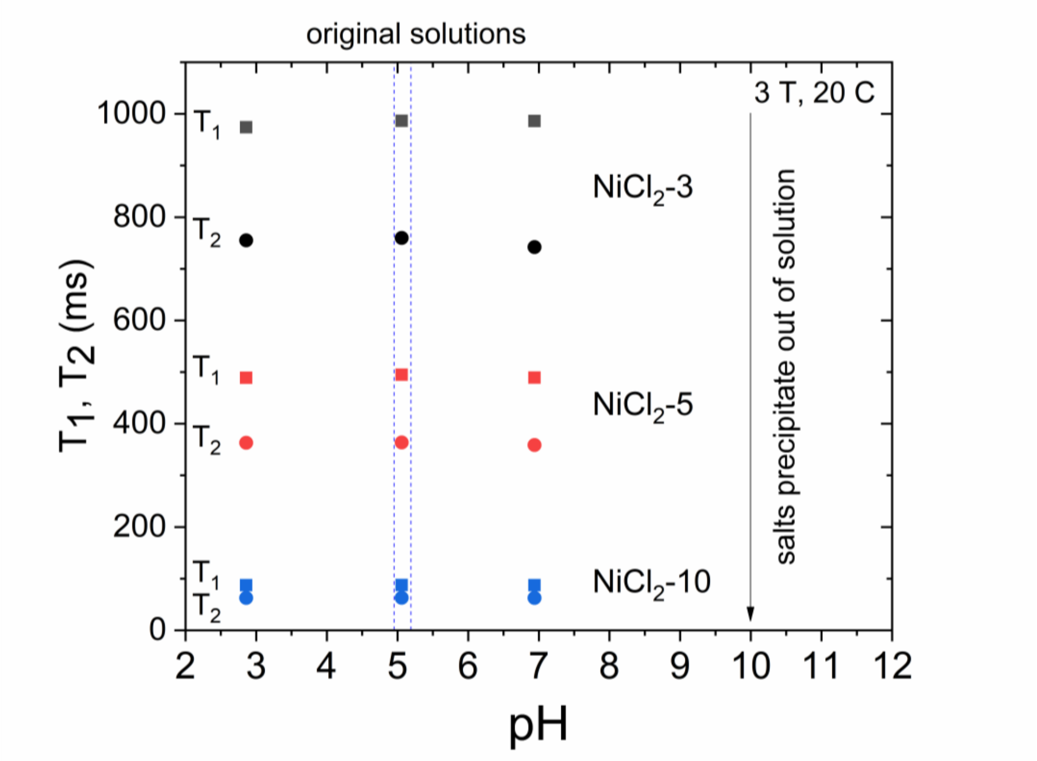

FIGURE S16 **Spin relaxation times for $NiCl_2$-3, $NiCl_2$-5, $NiCl_2$-10 at 3T, 20 °C as a function of pH.**

The water used for the solutions was ACS grade. Figure S17 shows a study comparing ACS grade water spin relaxation times to standard deionized water. The relaxation times were not sensitive to the grade of high purity water used as well as handling methods.

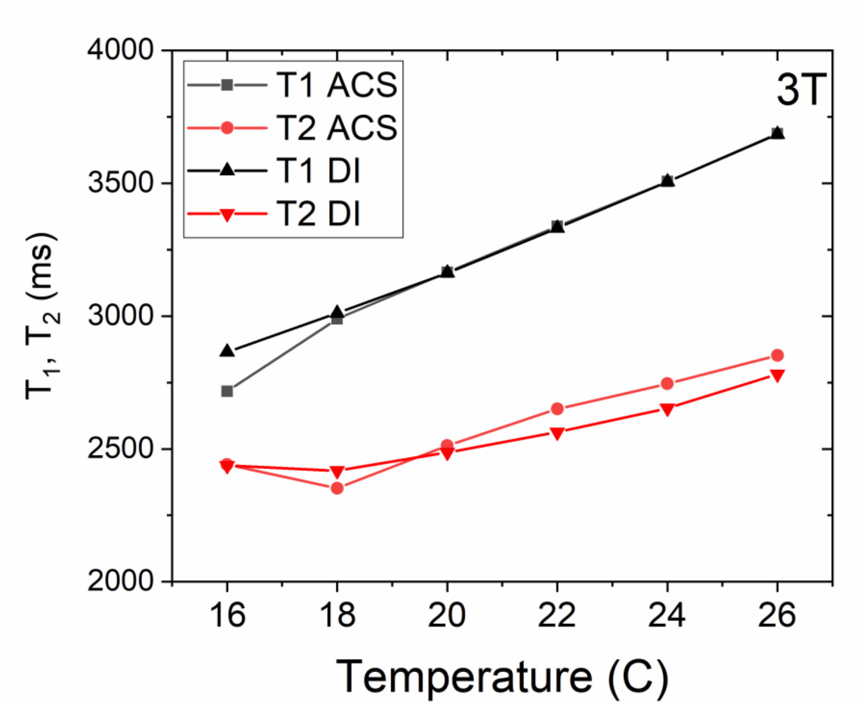

FIGURE S17 Relaxation times for ACS-grade and deionized water as a function of temperature.

9. The Commercial System Phantom

Two prototype system phantoms were manufactured by High Precision Devices (HPD), Boulder CO (https://hpd-online.com/). Subsequently, a company, CaliberMRI (https://qmri.com/), was formed (formerly QalibreMD, a subsidiary of HPD) to commercialize phantoms for quantitative MRI. The phantoms available include: the system phantom discussed in this work; a diffusion phantom covering the clinically relevant range of water diffusion coefficients; a breast phantom presenting with fat and fibroglandular tissue mimics, and cells with a range of T_1 values and diffusion coefficients to mimic healthy and tumor tissue; and a prostate phantom including diffusion, T_1, and T_2 arrays. The first three commercial phantoms were co-developed with NIST-Boulder MRI Group, ISMRM SQMR committee, the National Cancer Institute, Radiological Society of North America Quantitative Imaging Biomarkers Alliance, the Breast Imaging Research Group from the University of California San Francisco. The prostate phantom was co-developed with the group of Prof. Greg Metzger, University of Minnesota.

The initial design and implementation of the system phantom described in this paper have gone through several revisions and additions. Most notable are the change in the number of fiducial markers as well as the selection of proper materials and coatings to minimize the permeation of the aqueous solutions and tissue mimics. Further, *in situ* phantom temperature measurements were enabled by integrating a proprietary MR-readable liquid crystal thermometer(7), allowing for temperature correction of the T_1 and T_2 values using a lookup table provided by NIST calibration report. Other changes include

1. The polyphenylene sulfide structural components were used on the system phantom prototypes whereas the commercial phantom uses largely polycarbonate components to reduce cost.
2. The commercial system phantom uses polypropylene spheres glued to the plates for the MR-property arrays to reduce cost, whereas the prototype system phantom used spheres glued onto screw-in plugs so they could be readily changed or rearranged.
3. The shell on the commercial system phantom was made more compact allowing it to fit into 32-channel head coils, where the ear to ear spacing must be equal to or less than 196 mm.
4. Fiducial sphere # 56, directly under the fill port, was removed to allow access for a thermometer through the top fill port.

Different sets of solutions, with the same target concentrations, have been used in the commercial phantoms. The uncertainty and variability in the concentrations are often larger than the uncertainty in

the measured relaxation times. The calibration report for the appropriate phantom series, as determined from the serial number, should be used. An example SI-traceable calibration for commercial system phantoms that are part of the NIST/NIBIB Phantom Lending Library can be found at https://github.com/MRIStandards/SystemPhantom/blob/master/CalibrationData/800100S_SystemPhantomSolutions_Calibration_20200820_PLLSN0133_SN0134.pdf.

CaliberMRI has a beta version of a cloud-based software platform qCal-MR https://qcalsoftware.com/ that allows for fully automated measurements of all quantitative MRI parameters in the diffusion, breast and system phantoms, including auto-correction for the temperature. This software is distinct and separate from the open source code available at https://github.com/MRIStandards/PhantomViewer .

REFERENCES

1. Boss MA, Dienstfrey AM, Gimbutas Z, Keenan KE, Splett JD, Stupic KF, Russek SE. Magnetic Resonance Imaging Biomarker Calibration Service: Proton Spin Relaxation Times. 2018;Special Publication (NIST SP) - 250-97.
2. Berger S, Braun S. 200 and More NMR Experiments: A Practical Course: Wiley-VCH; 2004. 854 p.
3. Levitt MH, Freeman R. NMR Population-Inversion Using A Composite Pulse. J Magn Reson 1979;33(2):473-476.
4. Hahn EL. Spin Echoes. Physical Review 1950;80(4):580-594.
5. Carr HY, Purcell EM. Effects Of Diffusion On Free Precession In Nuclear Magnetic Resonance Experiments. Physical Review 1954;94(3):630-638.
6. Meiboom S, Gill D. Modified Spin-Echo Method For Measuring Nuclear Relaxation Times. Review of Scientific Instruments 1958;29(8):688-691.
7. Keenan KE, Stupic KF, Russek SE, Mirowski E. MRI-visible liquid crystal thermometer. J Magn Reson Imaging. 2016;44:610-619.
